# Modular Open-Source Stirred Tank Reactor (MOSTR), an Internet of Things (IoT) system for engineering education

**DOI:** 10.1016/j.ohx.2026.e00776

**Published:** 2026-04-24

**Authors:** Krys Bangert, Edward Browncross, Chalak Omar

**Affiliations:** Multidisciplinary Engineering Education, The University of Sheffield, The Diamond, 32 Leavygreave Road, Sheffield S3 7RD, United Kingdom

**Keywords:** Open-source, Chemical engineering, Education, Stirred tank reactor, IoT, Laboratory pedagogy

## Abstract

This paper presents the Modular Open-Source Stirred Tank Reactor (MOSTR), an IoT-enabled system developed for teaching chemical engineering principles such as residence time distribution, flow balancing and process instrumentation within higher education laboratories. Developed as a low-cost and pedagogically informed alternative to commercial units, the MOSTR’s modular design allows for the simultaneous deployment of multiple rigs to enhance student engagement and inclusivity in practical learning at scale. The reactor integrates an M5Stack-based control unit with flow, temperature, and conductivity sensing, supported by real-time data streaming via Wi-Fi and Virtual Machine (VM) server infrastructure. The design emphasizes reproducibility through accessible fabrication techniques, such as 3D printing and prioritises both maintainability and pedagogical alignment. Characterisation tests demonstrate that the system is highly sensitive to mixing variations and modular configurations, which highlights its versatility for diverse educational experiments. To support global adoption and adaptation in open engineering education, all design files, software, and documentation are shared under CC BY 4.0 and MIT licenses.

## Graphical abstract

**Figure:**
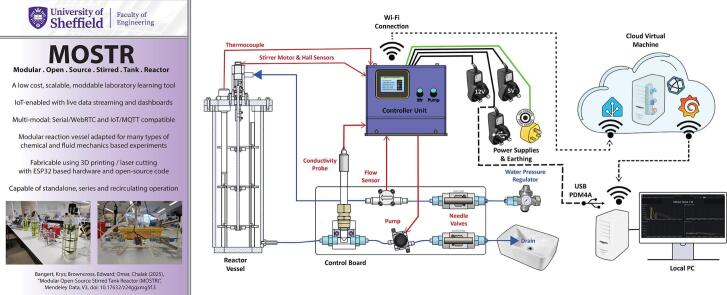

## Nomenclature

Parameter Notation: Parameter Definition (Unit)
D_t_: Vessel Width (m)
D_i_: Impeller Diameter (m)
H_i_: Impeller Distance from Vessel Base (m)
H_L_: Fluid Level (m)
W_i_: Impeller Paddle Height (m)
L_i_: Impeller Paddle Length (m)
W_b_: Baffle Width (m)
Q: Flow rate (L/min)
f: Hall pulse frequency (pulse/sec)
k: Flowmeter stock flow coefficient (Dimensionless)
K: Flowmeter nozzle specific coefficient (Dimensionless)
C_cond: Conductivity Reading (mS/cm)
t: Data logged time step / Time between measurements (sec, mins)
C(t): NaCl equivalent tracer concentration as a function of time (g/ml)
ΔN: Amount of tracer leaving the reactor (g)
v: Volumetric flow rate (ml/sec or L/min)
M: Amount of NaCl injected in tracer (g)
N_0_: Total amount of tracer used during the experiment (g)
$\overset{¯}{t}$: Mean Residence Time (sec)
τ (or τ_cuml_): Reactor Space Time (sec)
v_0_: Inlet volumetric flow rate (ml/sec)
V (or V_i_): Reactor working volume (ml)
S: Slope of the linear regression tail (sec^−1^)
⍺: Reactor Active Volume (Dimensionless (%))
β: Bypass flow fraction (Dimensionless (%))
E(t): Residence Time Distribution (RTD) per unit time (sec^−1^)
σ^2^: Statistical Variance (sec^2^)
n: Tanks In Series (TIS) one parameter model (Dimensionless)
Φ: Total Volume Utilisation (Dimensionless)
ρ: Density of water (kg/m^3^)
μ: Dynamic Viscosity of water (kg·m^−1^·s^−1^)
c: Impeller rotational speed (rev/sec)
Re: Reynolds Number (Dimensionless)
P: Power used by the impeller (Watts)
N_p_: Power Number coefficient (Dimensionless)
K_p_: Laminar Constant in N_p_ approximation (Dimensionless)
N_p∞_: Turbulent Constant in N_p_ approximation (Dimensionless)
m: Transition Factor in N_p_ approximation (Dimensionless)
∊_avg_: Average Energy Dissipation Rate (Watts/kg)
HH: Hydraulic Head in reactor vessel (cm)

Specifications table

**Table:** 

Hardware name	Modular Open-Source Stirred Tank Reactor (MOSTR)
Subject area	Educational tools and open source alternatives to existing infrastructure
Hardware type	Measuring physical properties and in-lab sensors
Closest commercial analog	*GUNT CE 320 Stirred Tank Reactor*
Open source license	● Hardware: *CC BY 4.0* ● Documentation: *CC BY 4.0* ● Software: *MIT Licence*
Cost of hardware	*Approx. £785 per rig (10 units fabricated for ∼£7851 total)*
Source file repository	● *Mendeley Data:* *https://doi.org/10.17632/z24ggxmg5f.3* Note: Software at time of release also available on the Mendeley Data repository. ● *Ongoing software developments available on GitHub:* *https://github.com/University-of-Sheffield-MEE/Modular-Open-Source-Stirred-Tank-Reactor*
OSHWA Certification UID	UK000088

### 1 Hardware in context

Practical laboratory education in chemical engineering traditionally relies on expensive, singular, large-scale research equipment to demonstrate physical principles. This reliance creates significant functional constraints: high student-to-equipment ratios limit direct “hands-on” experience, while substantial maintenance costs and restricted laboratory space further compound the issue [1]. Some institutions address this problem through centralised, high-funding facilities [2], smaller teaching spaces with limited resources require a more versatile, scalable approach to instrumentation.

The Modular Open-Source Stirred Tank Reactor (MOSTR) was developed as a networked alternative to democratise access to process engineering experimentation. Part of a wider initiative at the University of Sheffield to embed replicable educational resources across multidisciplinary curricula, the MOSTR replaces singular, high-volume commercial reactors with a suite of smaller, readily manufacturable rigs. Each unit is capable of independent or series operation and remote monitoring, supporting both in-person and blended (synchronous/asynchronous) learning models. Unlike commercial systems, which often prioritise proprietary IP and profit, the MOSTR design is optimised through a dual lens of engineering rigor and pedagogical principles.

The viability of rapid-prototyping and open-source methodology is increasingly supported by recent process chemistry and hardware literature. Foundational platforms like the “fReactor” have demonstrated that inexpensive, modular Continuous Stirred Tank Reactor (CSTR) designs can successfully democratise continuous-flow chemistry [3]. Building on this, researchers have utilized 3D-printed miniature CSTRs to safely handle complex gas–liquid-solid triphasic aerobic oxidations, proving that additive manufacturing can yield robust reactor environments [4]. Furthermore, the growth of documented, replicable open-hardware initiatives underscores a universal drive toward accessible laboratory infrastructure [5]. While existing efforts include microfluidic reactors and bioprocess teaching tools [6], [7], few are purpose-built for pedagogical contexts that emphasise learning analytics and IoT integration. The MOSTR addresses this gap by combining open digital infrastructure with educational alignment, safety compliance and comprehensive, reproducible documentation.

### 2 Hardware description

**Fig. 1:**
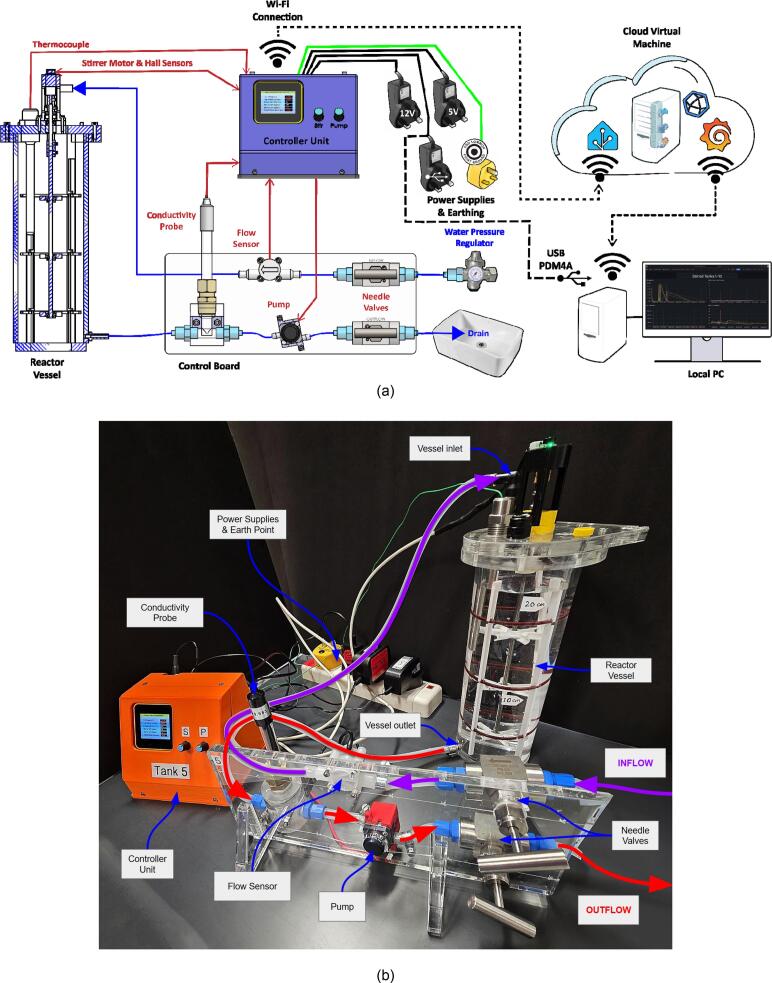
Overview of the MOSTR system; (a) diagrammatic representation, (b) single setup connected in the teaching laboratory. Purple arrows show water inflow and red arrows show water outflow. (For interpretation of the references to colour in this figure legend, the reader is referred to the web version of this article.)

A photographic and diagrammatic representation of an individual MOSTR system is shown in Fig. 1. The constituent physical sub-assemblies, controls arrangements and software are described in further detail in the subsequent sections.

The system design provides the following benefits:

● Reduced cost and increased scalability of laboratory-based learning.
● Provision of IoT-enabled live data and dashboards for interactive learning.
● Modular design, facilitating adaptation for other chemical, biochemical and fluid mechanics based experiments.
● Fabrication using readily available materials and open-source code.
● Alignment with constructivist pedagogical models by emphasising active experimentation and student-led investigation.

Broader Research Applications:

While this work primarily focuses on the MOSTR platform's capability for pulse-input Residence Time Distribution (RTD) analysis, the hardware suite, comprising continuous conductivity, temperature and high-resolution encoder telemetry, supports numerous standard and novel laboratory tasks:

● Batch Mixing & Reaction Kinetics: Without further modification the system natively supports mixing time analysis, step-response (F-curve) characterisation [8] and reaction kinetics studies, such as monitoring the saponification of ethyl acetate via conductivity decay [9].
● Fluid Dynamics and Hydrodynamic Prototyping: The transparent, unbaffled acrylic vessel permits visual dimensional analysis, allowing students/researchers to study different fluids regimes or correlate central vortex depth with the dimensionless Froude number across varying agitation rates [10].
● Process Control and Teleoperation Research: The digital infrastructure serves as a practical testbed for validating control theory; researchers/students can manipulate active Proportional–Integral (PI) parameters to analyse motor step-responses, system damping and load disturbance rejection directly through the telemetry interface, or utilise the WebRTC telemetry for testing low-latency remote operations.
● Scalable Multi-Stage Process Simulation: Because of its extremely low cost and networked architecture, researchers can easily deploy and monitor multiple reactor units in series or parallel. This facilitates the physical modelling of complex, multi-stage industrial flow-balancing processes without the high expense of commercial pilot plants.
● Customisable Sensor and Hardware Integration: The flexible header plate and I2C multiplexing system allow researchers from other disciplines (such as bioprocessing) to easily modify the control volume and integrate custom third-party sensors (e.g. pH or dissolved oxygen probes) for novel laboratory tasks.

#### 2.1 Reactor vessel assembly

The reactor assembly comprises two primary components: a flanged acrylic chamber with a 1 L working volume and a corresponding acrylic interfacing header unit. Acrylic was selected for the vessel because its optical transparency facilitates flow visualisation and pedagogical demonstrations, while its low cost ensures compatibility with standard laser-cutting fabrication. The process fluid (water) enters via an intake port in the header section and exits through a discharge port at the base of the 1 L chamber.

Mixing is driven by three miniature turbines mounted on a threaded bar, that is secured to a coupling and bearing assembly powered by a motorised gear system. To improve mixing efficiency, the design includes a free-standing, concentric baffle assembly positioned at the base of the chamber. For experiments sensitive to evaporation or contamination, the system can also be sealed using a silicone gasket, O-rings, and PTFE seals, plus a nitrile rubber radial seal within the bearing assembly to ensure an airtight fit.

As the experimental system was designed to operate with high salinity tracers (and other organisms/reagents), all hardware in direct contact with the working fluid is either made from plastic or 316 grade stainless steel to increase corrosion resistance. While polycarbonate, glass, or alternative metals may be substituted for specific chemical compatibility requirements, such modifications would necessitate more complex fabrication methods such as CNC machining or specialised glassblowing.

##### 2.1.1 Header plate

As with most reactor variants (Turbidostats, Bioreactors etc), the header plate is designed to be flexible and customisable for different experiments. The presented design has the following configuration:

● 2x ¼” BSP ports for push fit liquid pipe connection
● 1x ½” BSP port to house a thermowell & thermocouple
● 9x Ø4.5 mm holes, one for the stirrer shaft the rest for flange fixings
● 1x Ø2 mm hole for tracer injection
● 4x M3 tapped holes for the motor/bearing assembly mounting

**Fig. 2:**
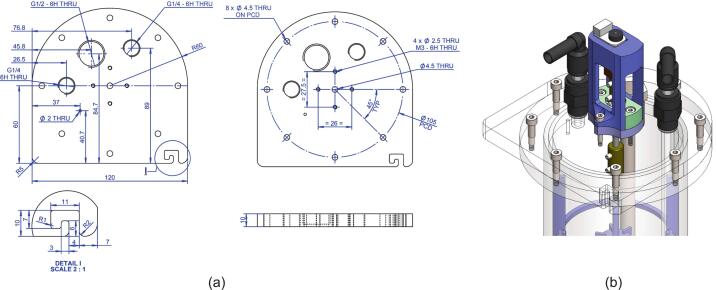
Overview of the header plate design. (a) Overall dimensions and port layout, (b) The plate, complete with various connectors.

This design leaves plenty of room for the inclusion of other sensors and ports by experimentalists and the thread standards used are quite common in fluid/gas interface connections (including low-cost domestic plumbing parts). In addition to the various holes, a wire retaining area (see Detail I of Fig. 2a) was incorporated to keep the motor controller and thermocouple wires attached to the lid using the “hooked” section of the header plate profile. Note: “G” thread equates to British Standard Pipe (BSP) thread.

**Fig. 3:**
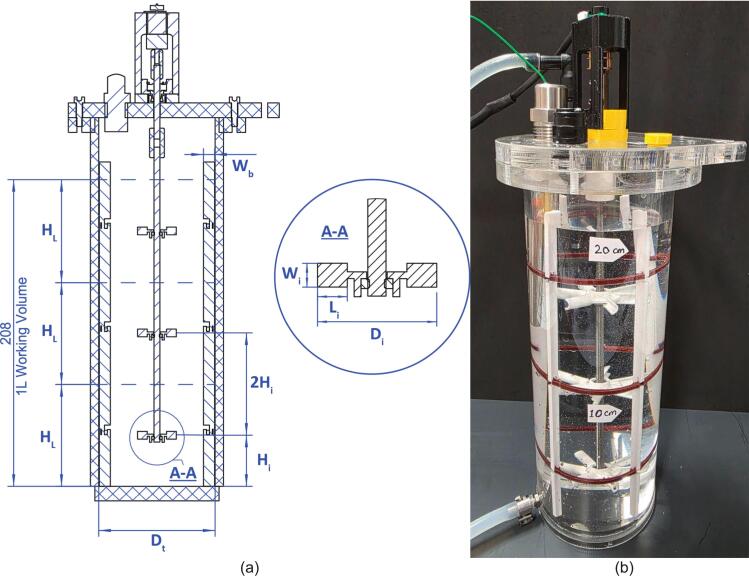
(a) Key reactor vessel & impeller dimensions based on Rushton design. (b) Close up of the reactor vessel.

##### 2.1.2 Reactor vessel

The system’s vessel geometry was governed by three factors; the requirement for a 1 L volume (to allow rapid tracer analysis, discussed later), interoperability with existing custom designs to increase cost efficiency via sharing vessel hardware between experimental setups [6] and to promote homogeneous fluid mixing. To ensure predictable hydrodynamic behaviour and facilitate direct comparison with theoretical models, the vessel, baffle, and impeller geometries were proportioned according to the classical standard configurations established by Rushton et al [11]. Utilising these foundational design ratios allows the system to leverage well characterised power and flow correlations, providing a reliable baseline for comparative tests. In this configuration (Table 1), a standard mixing vessel maintains a liquid height equal to the tank diameter (H_L_ = D_t_) and an impeller diameter one-third of the tank diameter (D_i_ = D_t_/3), which establishes the conventional H_L_/D_i_ baseline of 3 to optimally balance top-to-bottom circulation. The only deviation from convention in this design setup was due to the geometric ratio of the acrylic tube ID in relation to the 1 L fill level; this was to keep costs down by utilising supplier common sizes and maintain compatibility with other pre-existing teaching equipment. The effects of this variance resulted in a slightly compressed H_L_/D_i_ ratio of 2.66, which was deemed to be an acceptable compromise. The impeller placement in each of the three working volume regions is governed by *H_L_ / 2,* which is common practice and also within the window of optimisation of mixing and power draw based on impeller spacings of 2 to 3 multiples of the D_i_ [12]. The threaded shaft design allows for the adjustment or removal of impellers to suit various experimental permutations. Users may also modify the overall working volume by increasing the acrylic tubing height and adding impeller turbines or scale the system using the standard chemical engineering empirical design constants (Table 1).

**Table 1:** Typical vessel to Rushton impeller design ratios, where: D_t_ = Vessel Width, D_i_ = Impeller Diameter, H_i_ = Impeller Distance from Vessel Base, H_L_ = Fluid Level, W_i_ = Impeller Paddle Height, L_i_ = Impeller Paddle Length, W_b_ = Baffle Width.

Impeller					Baffles	
D_t_/D_i_	H_L_/D_i_	H_i_/D_i_	W_i_/D_i_	L_i_/D_i_	W_b_/D_t_	Num
3	3	1	0.2	0.25	0.1	4

#### 2.2 Inlet/outlet manual flow control board and conductivity sensor

**Fig. 4:**
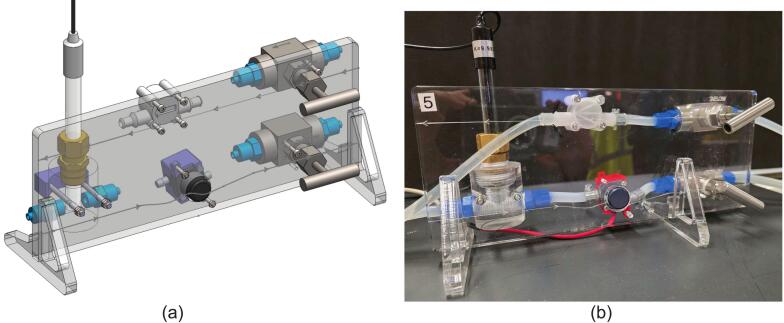
Overview of the control board design. (a) CAD Assembly. (b) Actual board complete with various connectors.

The inflow/outflow of water through the apparatus is manually controlled using needle valves mounted on a laser cut acrylic control board. Each valve is connected by flexible PVC/silicone pipes (see Fig. 4b). The inlet needle valve is connected to a laboratory water tap that is pressure regulated; the output of the valve then connects in series to a 508–2704 RS PRO flow meter (configured with a 1 mm nozzle for a flow range of 0.05–0.5 L/min) and then the inlet of the reactor vessel. The outlet of the vessel at the base is connected back to the board starting with the DFR0300-H DFRobot conductivity probe, then into the 702–6882 RS PRO direct coupling centrifugal water pump and finally another needle valve before the water enters the drain.

The experiment setup is designed so that continuous manual input is required to simulate an industrial calibration scenario. The inflow and outflow of water must be balanced as equally as possible, to ensure that the experimental control volume is constant (approx. 1 L), with a steady state inflow of fresh water and outflow of tracer and stirred water. This mimics a typical setup for a CSTR process in industrial chemical engineering. The apparatus is highly sensitive to adjustments due to the inclusion of the needle valves. The addition of the inline pump on the exit also allows the system to have higher flow throughputs than driven by the hydrostatic head of the tank alone. In addition, the pump allows the system to operate in a self-recirculating configuration (rather than draining) and in a modular manner to connect multiple setups in series to conduct experiments on multistage CSTRs.

**Fig. 5:**
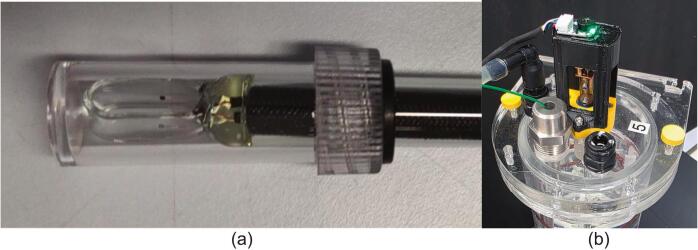
(a) Conductivity probe end, (b) Motor encoder assembly and thermowell.

The flow meter transmits a series of hall sensor digital pulses to the STM32F030 M5Stack encoder in the controller unit (CU), this data is then interpreted and displayed locally on the CU’s LCD as flow rate in L/min. The pump is controlled by PWM output from a U160-V11 M5Stack H-Bridge unit in the CU, the amplitude of which is controlled by a potentiometer (M5Stack U005) on the CU box. The corresponding “Pump Power” value ranging from 0 to 100 %, is also displayed on the LCD. The conductivity probe is retained in a custom machined acrylic flow cell using a compression fitting with a Viton O-ring seal. This allows the probe's hooped tip to be aligned so the conductivity pads are parallel to the water flow (see Fig. 5a) and to allow for the calibration of the probe and the purging of trapped air upon each extraction. The sensor signals are interpreted by the DFR0300-H control board in the CU and shown on the LCD as values of ms/cm. In addition to being displayed on the CU display, the data is also transmitted using Message Queuing Telemetry Transport (MQTT) protocols for Internet of Things (IoT) communication over Wi-Fi, to a Virtual Machine (VM) hosted instance of Home Assistant (HA).

#### 2.3 Stirrer assembly, rotary sensor & thermowell

A 6 V DFRobot FIT0483 Geared Motor with integrated hall effect encoder provides stirring control to the reactor system. The motor is mounted within a modular 3d printed housing that also integrates a bearing, rotary seal and coupling (see Fig. 5b). The design allows direct access to the retaining grub screw, facilitating rapid motor swaps or gearing adjustments without requiring a full teardown of the assembly. Additionally, a separate coupling within the reactor chamber enables the use of various low-cost, 3D-printed impeller arrangements.

The stirrer motor and encoder are connected to the CU via a single cable, which interfaces with a U160-V11 H-Bridge and an STM32F030 encoder module. This cable carries both power and dual-directional control signals. Similar to the pump configuration, a potentiometer (M5Stack U005) on the CU allows the user to define a rotation setpoint, which is displayed on the local LCD. A software-based PI controller then modulates the PWM output to align the stirrer speed with the specified value, utilising feedback from the encoder's Hall effect sensors. Both the target setpoint and the measured rotational speed are displayed on the LCD and streamed via Wi-Fi to the Home Assistant interface, consistent with the telemetry protocol used for the conductivity probe.

The water temperature is monitored using a Type K thermocouple, which is housed within a 316-grade stainless steel thermowell attached to the vessel lid. This stainless steel construction ensures the unit can withstand the corrosive effects of the high-salinity tracers used during experimentation. The use of a thermowell also provides the flexibility to interchange different thermocouple types without requiring structural modifications to the reactor. The Type K probe interfaces with a U133-V11 M5Stack KMeter-ISO unit within the CU; as with the other sensors, the real-time readout is displayed on the local LCD and transmitted over Wi-Fi. Additionally, these thermal data points are utilised during the conductivity probe calibration process (see Section 6.2).

#### 2.4 Modular ESP32 control hardware

**Fig. 6:**
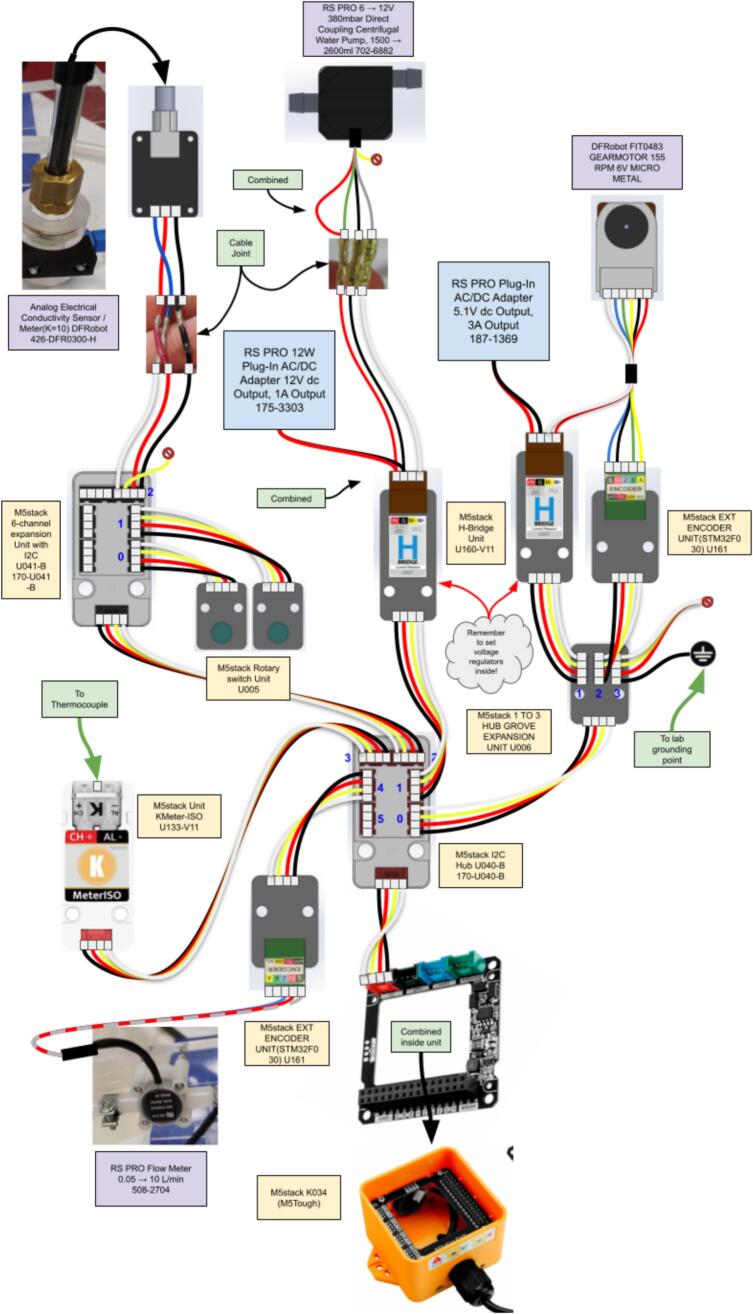
A diagrammatic representation of the MOSTR system hardware connections.

**Fig. 7:**
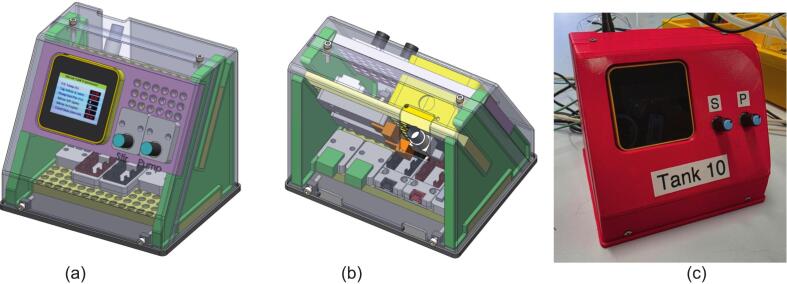
MOSTR system Control Unit assembly; (a) / (b) CAD representation of the CU internal layout. (c) Photo of the actual unit.

The system is designed to be modular in software design and hardware. The computation is handled by a low-cost ESP32 chipset based microcontroller (M5Tough): part of M5Stack’s ecosystem of products. To allow full modularity and subsequent further expansion, all sensors (again with M5Stacks’s form factor) are multiplexed via the I2C protocol (see Fig. 6). For sensors that are not I2C compatible (the rotary potentiometers and conductivity unit), an I2C 6-channel GPIO expander unit is used to make the data addressable. The decision to use the M5Stack system of products was to allow swappable control modules without the need for custom circuits and soldering of individual components. This approach, although marginally more expensive than designing from scratch, has the benefits of allowing rapid construction, maintenance and modification, which is highly beneficial for educational equipment that is used for mass teaching in undergraduate laboratories. Even users with little understanding of micro-electronics can fault-find these systems, by simply swapping out I2C modules systematically (Fig. 7).

To avoid the need for complex power management systems, U160-V11 M5Stack H-Bridge units are connected to simple 5 V and 12 V supplies that power the motor and pump systems. These units are simple mains voltage transformer modules with low voltage outputs, inherently safe compared to typical DIN-rail commercial systems. The M5Tough controller also has a direct USB connection wire that can also be used for power and data transfer if required. When operating in a standalone configuration, a typical domestic USB power supply will work.

As the power supplies used in the design are low voltage and without earth requirements, the system can have a “floating earth”, which can cause issues with conductivity readings. For this reason, one of the I2C connections is connected to an ESD Earth Bonding Plug that provides a local, safe ground reference. However, it is advised to consult your institution, local regulations, and/or a licensed technician for advice regarding power connections, regardless of requirements post-stepdown transformers.

#### 2.5 Control unit housing

The control unit housing has been designed to be completely 3D printed and held together with heat fused threaded inserts. Inside the enclosure each of the M5Stack modules (and conductivity probe board) are mounted on one of three boards. The boards themselves are designed to replicate the Lego Technic push-fit mounting system that is also utilised by M5Stack. This makes changing hardware modules extremely easy and facilitates future cross compatibility with other projects and Lego based designs.

Accessibility cutouts are situated on the front panel to accommodate the potentiometer controls, LCD, conductivity probe and thermocouple interfaces. Additional cutouts are located on the rear base for the primary sensor/motor connections and power supply inputs.

#### 2.6 Modular software & data handling

**Fig. 8:**
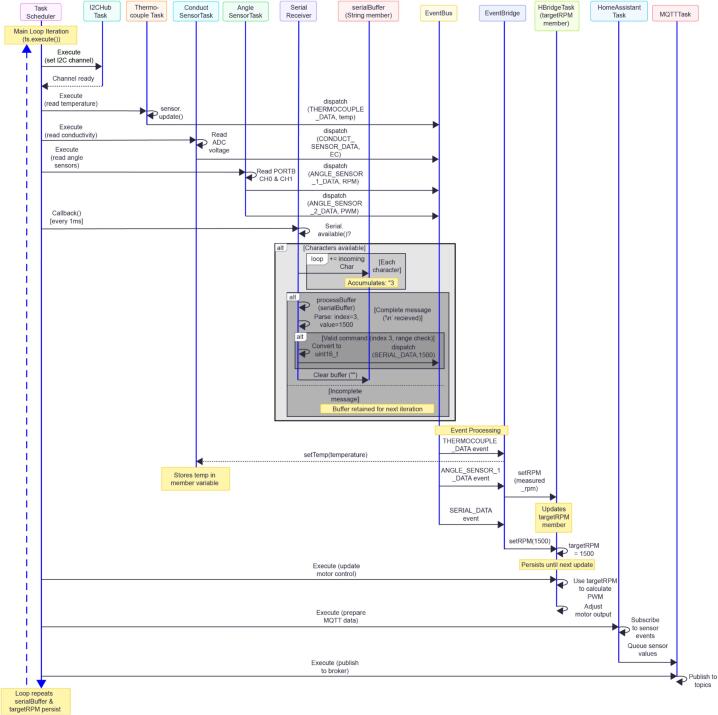
MOSTR system code execution sequence diagram. Top to Bottom: Time/Execution Flow; Represents the chronological progression of events during one scheduler cycle, each horizontal arrow represents an action/message that occurs after the previous one demonstrating how events propagate through the system over time. Left to Right: System Components/Actors representing different software modules/objects in the system. Key Visual Elements: Solid arrows: Synchronous calls/dispatches, Dashed arrows: Returns or asynchronous responses, Boxes over lifelines: Notes about internal state or actions, Alt/Loop boxes: Conditional logic or repeated operations Notes: Contextual information about timing or state persistence.

The system's software is coded in the C++ programming language based around a series of modular libraries that are adapted from freely available Arduino code. This program runs on the M5Tough microcontroller and is organised around a software Task Scheduler plus an Event Bus (see Fig. 8). The main program enables a set of hardware tasks (I2C multiplexer “PaHub”, PortBHub ADC, thermocouple task, flow sensor, encoders, H‑bridge drivers, renderer, Wi‑Fi/MQTT/Home Assistant tasks, etc).

Each hardware interface is wrapped in a Task object that periodically runs and posts events onto the event bus. Consumers (for example the H‑bridge controller or the MQTT task) subscribe to those events and react to them as they occur. Persistent settings (such as Wi-Fi login/passwords and unit identifier names) come from an EEPROM-backed config file; MQTT and Home Assistant integration publish sensor values and receive commands. The main program loop executes the scheduler so each task can run at pre-configured time intervals.

**Fig. 9:**
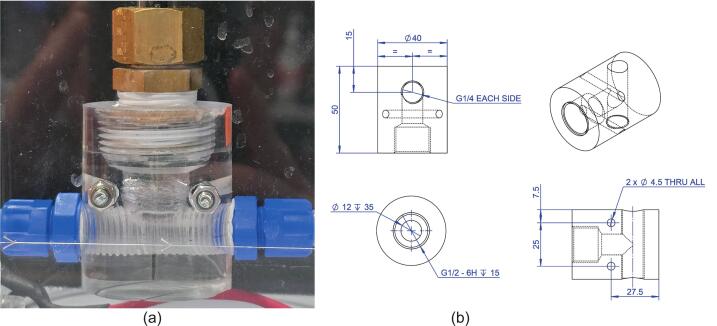
(a) Close up of the Conductivity probe adapter section. (b) Technical drawing of the acrylic section.

On startup the firmware shows an initial decision UI on the M5 touchscreen display that lets the operator calibrate the conductivity probe. The calibration screen temporarily performs low‑level I2C reads of the thermocouple and analogue reads from the PortB ADC to display live temperature and conductivity values. The calibrated temperature value is passed to the DFRobot_EC10 library for temperature compensation. Buttons on the screen invoke the EC calibration commands before continuing to normal operation, where sensor readings and control loops run under scheduled tasks and publish through MQTT/Home Assistant (Fig. 9).

The development environment used for programming is Visual Studio Code with the PlatformIO extension, which manages the ESP32-based M5Tough build toolchain. PlatformIO handles library dependencies (M5Tough, TaskScheduler, EventBus, DFRobot_EC10, MQTT, etc.) defined in platformio.ini, compiles the C++ source files using the Arduino framework for ESP32, and links them into a single firmware binary. When “Upload” is triggered, PlatformIO invokes esptool.py to flash the compiled.bin file over USB serial to the M5Tough's flash memory; the device then boots into the new firmware. Serial monitor functionality in VSCode allows real-time debugging output from the running microcontroller.

Sensor data from the M5Tough are averaged and sent every 5 s over Wi-Fi, via an MQTT broker, to Home Assistant (utilising the Home Assistant MQTT integration). The Home Assistant MQTT integration can accept special MQTT messages called discovery messages to register or update new devices and their sensors. The firmware sends out such a discovery message on each startup.

Home Assistant and a Mosquitto MQTT server run in Docker containers on a virtual machine. Home Assistant is configured to persist historic sensor data in an InfluxDB instance (also containerised on the VM). This stores conductivity, temperature, flow rate, and RPM readings with timestamps indefinitely. Grafana dashboards, containerised on the VM, provide real-time visualisation of the InfluxDB data via customizable panels, diagrams, and tables. The interface supports time-range zooming, data filtering, and CSV export for offline analysis. This modular architecture separates data collection (M5Tough), orchestration (Home Assistant), persistence (InfluxDB) and visualisation (Grafana) into distinct services, allowing individual components to be swapped to meet specific research requirements.

The SerialReceiverTask section of the code also implements a lightweight, extensible serial communication protocol that serves as the hardware abstraction layer between the M5Tough and potential external control systems, particularly web-based remote laboratory interfaces using WebRTC technology [13]. When the M5Stack is connected directly to a PC/laptop/RaspberryPI or similar, it continuously polls the USB serial port, accumulating incoming characters into a buffer until a newline delimiter is received. The protocol uses a simple text-based format (<index>#<value>\n) where the index identifies the command type and the value contains the associated parameter, minimising communication overhead for low-latency real-time control as recommended in remote laboratory architectures.

The implementation currently supports two primary command types: device identification (index 0) which responds with the device name to enable automatic enumeration of multiple connected MOSTR systems, and motor speed control (index 3) which validates incoming RPM values (0–65535), converts them to 16-bit integers, and dispatches them as events onto the EventBus for processing by motor control tasks. This design enables seamless integration with WebRTC DataChannels where browser-based interfaces can send commands that traverse peer-to-peer connections, through the local computer's USB serial port, to the M5Stack hardware with end-to-end latency under 200 ms. The protocol is easily extensible, and new command indices could be added for, for example, temperature control, pH setpoints, emergency stops, or other experimental parameters, without modifying the underlying communication framework, supporting the scalability and flexibility requirements of multi-user remote laboratory systems for practical education and research applications.

##### 2.6.1 Serial/WebRTC vs. IoT/MQTT approaches

The system's dual-mode architecture provides complementary communication pathways that serve different use cases and operational requirements (see Fig. 8). The Serial/WebRTC approach excels in scenarios requiring ultra-low latency remote control, such as online educational laboratory sessions where students need immediate feedback from equipment manipulation [13]. With end-to-end latency under 200 ms and peer-to-peer connections that bypass intermediary servers, this method is ideal for real-time remote experiments or live demonstrations from lectures, provided USB connectivity and a computer capable of receiving WebRTC commands is available within the lab. The simplicity of the serial protocol (<index>#<value>\n) means minimal parsing overhead, deterministic behaviour, and easy debugging through serial terminal monitors. Additionally, the direct connection USB provides is more robust than Wi-Fi, making it highly reliable for critical control operations.

In contrast, the IoT/MQTT approach through Home Assistant, InfluxDB, and Grafana provides enterprise-grade data logging, multi-device orchestration and remote accessibility from anywhere on the network or internet. This architecture is superior for long running or steady state experiments requiring historical data analysis, automated alerts, and integration with building management systems or institutional infrastructure. MQTT's publish-subscribe model allows multiple consumers (mobile apps, dashboards, automated scripts) to simultaneously monitor the same MOSTR without additional device overhead, while InfluxDB provides time-series data retention with sub-second resolution for weeks or months of continuous operation. The web-based Grafana dashboards enable researchers to monitor experiments from home, export CSV datasets for statistical analysis and set up threshold alerts that trigger notifications without requiring constant supervision. However, this approach introduces higher latency (typically 500 ms − 2 sec) due to network hops, MQTT broker processing, database writes and requires stable Wi-Fi connectivity along with properly configured network infrastructure.

The MOSTR control system’s hybrid capability of supporting both modes simultaneously provides exceptional modular flexibility. With further development, in active teaching sessions, instructors could use the Serial/WebRTC interface for responsive student interaction while the IoT stack continues logging all sensor data in the background for post-experiment analysis. Researchers could remotely monitor long-term experiments via MQTT dashboards while retaining the ability to connect directly via USB for immediate troubleshooting or emergency intervention without network dependencies. This dual-mode design can also enable multiple layers of redundancy e.g. if the Wi-Fi network fails, local USB control remains functional and conversely, if the USB host computer is unavailable, the system continues autonomous operation with MQTT-based remote monitoring. The architecture effectively bridges the gap between real-time control systems and IoT data platforms, making the MOSTR or any other system using this multiplexed Arduino style hardware suitable for both hands-on educational environments and unattended research operations.

### 3 Design files summary

A complete inventory list of system fabrication and design files associated with the project are included in Table 2 grouped in subcategory folders as they are stored within the “Design Files” section of the online repository (https://doi.org/10.17632/z24ggxmg5f.3). All parametric 2D/3D SolidWorks (Version 2024 SP 5.0 Student Edition) and associated.DXF CAD files are included to facilitate customisation. To aid with 3d printing, .STL versions of the CAD geometry design intended for that mode of fabrication are also made available. For the conductivity probe interface and other laser cut parts that require further manual fabrication operations, a full set of 2D technical drawings are also included. Further instructions on these details are included in section 5. The software instance is provisioned in /Software of the https://doi.org/10.17632/z24ggxmg5f.3 repository (Latest at: https://github.com/University-of-Sheffield-MEE/Modular-Open-Source-Stirred-Tank-Reactor).

**Table 2:** Summary of the design related file locations for the MOSTR system. All design files are available at https://doi.org/10.17632/z24ggxmg5f.3, all with the open-source licence CC BY 4.0.

Content	File type(s)	Folder
Bill of materials (BOM) and ordering spreadsheet	.XLSX	Design Files/Bill of materials
All SolidWorks 2024 Files for the project, including 3D & 2D.	.SLDASM,.SLPRT,.SLDDRW	Design Files/Solidworks
All 2D Path files needed for laser/waterjet cutting.	.DXF	Design Files/DXFs
All 3D Printable Geometry for the project.	.STL	Design Files/STLs
All 2D drawings for the project in PDF format for printing.	.PDF	Design Files/Technical Drawings PDFs
A PDF of the wiring connections required.	.PDF	Design Files/Wiring Diagram

### 4 Bill of materials summary

The ‘MOSTR_Bill_Of_Materials_r2.xlsx’ file (https://doi.org/10.17632/z24ggxmg5f.3) provides a comprehensive Bill of Materials, including specific part numbers, suppliers and quantities. Table 3 details the total cost breakdown for each sub-assembly. The largest proportion of project costs is attributed to the calibrated sensors and pump units; however, the total cost per system (£785.16) is an order of magnitude lower than typical commercial equivalents while offering significantly greater adaptability.

**Table 3:** Overview of estimated costs to make a single MOSTR system. The costs are grouped by major sub assembly, with approximations made for manufactured items where bulk materials were required (i.e. acrylic tube), alongside unit costs such as individual components. All prices are in UK sterling equivalent.

Grouping	Total Cost (£)
MOSTR Control Unit SA	275.33
MOSTR Stirred Tank and Head SA	104.61
MOSTR Valve Board SA	405.21
Grand Total	785.16

### 5 Build instructions

#### 5.1 Stirred tank and head SA

##### 5.1.1 Acrylic fabrication

The reactor vessel and header plate consist of four sections made from transparent acrylic plastic; the base, interface flange (MOSTR_Main_Body_1.dxf) and header (MOSTR_Lid_1.dxf) are laser cut from 10 mm sheet and the main body of cut sections of 90 mm OD, 78 mm ID cast tube. The laser cut flange and base sections were then further processed by turning on a lathe to create a stepped tolerance fit that facilitated acrylic bonding with adhesive. The .DXF files have incorporated tolerance to facilitate this post-cut machining sequence. Note: It is highly recommended that leak tests are performed following the bonding stage. The final step was to tap threads into the flange and lid sections ready for the various fittings. Details on the dimensions and threads required are shown on technical drawings: MOSTR_Main_Body_1.pdf and MOSTR_Lid_1.pdf. Laser cutters have the potential to cut in a non-parallel fashion through thicker materials, to mitigate this perform test cuts first to verify there is sufficient material left to apply threads, if not scale the holes accordingly. Tap the acrylic parts with machining oil, especially if using drills rather than hand tapping. The final parts to be laser cut in the SA are the four baffle plates, these are out of 3 mm thick acrylic sheet (MOSTR_Rushton_Turbine_Baffles_1.dxf). The speed and power associated with the cut will need to be optimised again to ensure accurate dimensional tolerances.

##### 5.1.2 3D printing

The reactor header has three 3D printed parts to form the motor and bearing sub-assembly, the dimensions for these are shown in MOSTR_Motor_Bearing_Housing_1_Sht_1-3.pdf. The bearing block (MOSTR_Motor_Bearing_Housing_1_-_Bearing_Block.stl), space block (MOSTR_Motor_Bearing_Housing_1_-_Spacer_Block.stl) and the motor mount (MOSTR_Motor_Bearing_Housing_1_-_Motor_Mount.stl). When printing use standard PLA filament and 0.4 mm nozzle arrangement, there is minimal need for additional support when sliced. Depending on the gear motor purchased, the motor mount may need to be adjusted to ensure a tolerance fit.

In the reactor vessel there are 6 further 3d printed elements, 3 baffle alignment rings (MOSTR_Rushton_Turbine_Baffles_1_-_Baffle_Ring.stl) and 3 Rushton impellers (MOSTR_Rushton_Turbine_1.stl). The dimensions for these are shown in MOSTR_Rushton_Turbine_1.pdf and MOSTR_Rushton_Turbine_Baffles_1_Sht_1-2.pdf. Scale the SolidWorks impeller geometry as required using the embedded parametric table within the file, following the ratios discussed in Section 2.1.2. Use a standard PLA filament and 0.4 mm nozzle arrangement. The Rushton part will require additional support when sliced. Print the baffle ring with a 0.2 mm nozzle to assist with the mating parts (see Detail II in the associated drawing).

##### 5.1.3 Other operations

The SA (MOSTR_Stirred_Tank_and_Head_SA_1.pdf) also requires the following items and operations:

● 1x 50 mm long section of 316 ST/ST Rod Ø4 mm rod, cut to length and deburred (Item 4).
● 1x 200 mm long 304 ST/ST M4 Threaded Rod cut to length, deburred then re-threading at the end with a die (Item 14).
● 2x 8 mm OD x 2 mm thick Clear Acrylic Tube cut to length (50 mm to 270 mm long depending on experimental requirement) and deburred (Items 17/18).
● 2x Push-fit Threaded Adaptor, ¼” BSP Male to Push In 8 mm, drilled through with a 9 mm drill bit to accommodate the 8 mm OD acrylic tube, which is then inserted through into the vessel (Item 16).

##### 5.1.4 Assembly

Follow this sequence to assemble the header plate (see MOSTR_Stirred_Tank_and_Head_SA_1.pdf, Item numbers in table):

1. Push the Nitrile Rubber Shaft Seal (Item 10) into the Spacer Block (Item 8)
2. Push Ball Bearing (Item 6) into the Bearing Block (Item 5), then optionally retain with the Circlip (Item 7).
3. Place and align the assembled Spacer Block on the Header Plate (Item 3), then mount the Bearing block on top. Fix this down with two of the M3 x 16 mm Cap Head Screws (Item 24) through the top of the bearing block into the Header Plate.
4. Push the 50 mm long section of Ø4 mm Rod (Item 4) through the bearing and seal so it protrudes through the other side of the plate. Add some silicone grease or other form of lubricant during the process on the shaft, if it is stiff pushing against a tabletop or tap gently with a rubber mallet if necessary.
5. Attach the In-line Coupling 3 mm − 4 mm (Item 11), 4 mm bore down, to the shaft above the bearing with one of the M3 x 6 mm grub screws (Item 26).
6. Place the Gear Motor and Encoder unit (Item 12) into the 3d printed Motor Mount (Item 9).
7. Align the motor and mount with the bearing block and In-line coupling and attach to the header plate with the remaining two M3 x 16 mm Cap Head Screws (Item 24). Note: the flat on the motor shaft must be aligned to be accessible through the side of the enclosure for the next step. If the motor is being forced up during the screwing process, the 4 mm shaft (Item 4) will need to be lowered slightly further.
8. Attach the In-line coupling to the motor shaft with one of the M3 x 6 grub screws (Item 26).
9. Screw in the Thermowell (Item 19) and both ¼” BSP Male to Push In 8 mm Straight Threaded Adaptors (Item 16), to ensure a good seal, add PTFE tape to the threads before attachment.
10. Push the 8 mm OD x 2 mm thick Clear Acrylic Tubes (Items 17/18) through the adapters (Item 17) to the required depth. Then push the 8 mm Elbow Tube-to-Tube Adaptors (Item 15) onto the tubes above the header plate.
11. Attach the Straight Threaded Adaptor (Item 20) outlet to the tank main body (Item 1) again with PTFE tape.
12. Begin to assemble the impeller section by adding the M4 Thin Nuts (Item 25) to the underside of all three Rushton impellers (Item 2). Each impeller is held in place with a nut either side of it when it is mounted on the threaded rod (Item 14). To get the correct spacing (see section 2.1.2), start by attaching the first impeller at the end of the rod, then add the rest sequentially by measuring the distance between them.
13. Attach the 4 mm − 4 mm In-line Coupling (Item 13) to the Ø4 mm Rod (Item 4) protruding through the header plate with a M3 x 6 mm Grub Screw (Item 26), before sliding the impeller section that is now attached to the M4 Threaded Rod (Item 14) into the other part of the coupling. Retain as before with another M3 x 6 mm grub screw.
14. The baffle assembly can then be constructed using Items 21 and 22. Slide each of the plates in sequence into the retaining rings. This is quite a delicate operation, depending on the quality of the 3d print and laser cutting. The retaining flaps of the ring may need to be manipulated using the end of a small flat bladed screwdriver or tweezers and the prints cleaned with a sharp blade before correct seating is achieved.
15. The final operation is to slide the baffle assembly into the vessel, then attach the header plate assembly to the vessel using the M4 x 16 mm Cap Head Screws (Item 24).

#### 5.2 Valve board SA

##### 5.2.1 Acrylic fabrication

As with the reactor vessel and header plate, the backing board (see MOSTR_Backing_Board_Sht_1-2.pdf) is made from 10 mm transparent acrylic plastic sheet. The .DXF files included (MOSTR_Backing_Board.dxf, MOSTR_Backing Board_Stand.dxf) have undersized holes to allow the cutting of threads as shown on the MOSTR_Backing_Board_Sht_1-2.pdf drawing. Perform test cuts and employ the techniques as mentioned in section 5.1.1. The .DXF includes both the contours for the backing board and the two stands for it, note that some of the cutting lines are for engraving to show the objects mounted and the flow of water to the apparatus, so the laser intensity and speed will need to be adjusted accordingly.

The conductivity probe adapter was made from an acrylic bar of Ø50 mm (see MOSTR_Probe_Adapter_-_Acrylic_Part_1.pdf) and has several fabrication steps; Firstly, to enable machining if the bar was drawn rather than cast, stress relieve it by annealing in a temperature-controlled oven. The program for this is 2 hrs 30 min heating to 140 °C, hold for 11 hrs 15 mins, cool to 110 °C over 5 hrs, then HOLD at 110 °C for 6 hrs, then cool to ROOM TEMP at 4 °C/hr for 24 hrs.

In a lathe, the bar can then have the Ø12 mm hole centre drilled, followed by a ½” BSP thread cut. The bar can then be drilled through on a pillar drill and tapped with a ¼” BSP at each side. The final operation is to drill the two Ø4.5 mm mounting holes perpendicular to the previous set of cuts. The fabrication process is very sensitive to feeds and good levels of lubrication due to the brittle nature of the acrylic. Use machining oil at every step, employ slow feeds and perform further annealing if required after major machining operations to relieve material stress. If lathe machining and annealing facilities are not available, a similar interface can be fabricated from threaded BSP pipefittings consisting of a ½” BSP female “T”, with two ½” BSP to ¼” BSP reducers. However, this would make the presence of air pockets not visible and a greater stagnant volume around the probe, which could lead to buffering effects.

##### 5.2.2 3D printing

Two other parts on the valve board assembly are 3d printed (see MOSTR_Motor_Lid_1.pdf and MOSTR_Probe_Adapter_-_3DP_Retainer_Part_1.pdf); the Motor Lid (Item 5, MOSTR_Motor_Lid_1.stl) and the Probe Adapter Retainer (Item 16, MOSTR_Probe_Adapter_1.stl). Use a standard PLA filament and 0.4 mm nozzle arrangement, the Motor Lid part will require additional support when sliced.

##### 5.2.3 Other operations

The SA also requires the following (see MOSTR_Valve_board_SA_1.pdf, Item numbers in table):

● Drill out the main body of the Brass Straight Compression Fitting (Item 5) to accommodate the diameter of the conductivity probe (Item 6). Maintain a slight clearance fit of approximately 0.5 mm to ensure a proper O-ring seal during assembly.

##### 5.2.4 Assembly

Follow this sequence to assemble the valve board:

1. Attach the ¼” BSP Push-on adapters (Item 7) to the needle valves (Item 9) and the acrylic probe adapter (Item 4), using PTFE tape to seal the threads.
2. Screw on the ½” BSP straight compression coupler (Item 5) to the acrylic probe adapter (Item 4) using PTFE. As this is a tapered thread, do not overtighten as this could fracture the adapter. When inserting the conductivity probe (Item 6) into the compression fitting, discard any domestic pipe sealing olives and replace with a silicone O-ring capable of creating a water-tight seal. A 2 mm thick Viton type should be used due to its chemical resistance.
3. Screw each of the M4 x 65 mm threaded rods (Item 11) into a M3 thin nut (Item 15), then slide the nuts into the probe adapter retainer (Item 16).
4. Mount the probe adapter assembly onto the threaded bars (Item 11) and slide it into place against the retainer. Feed the threaded rods through the backing board (Item 1) and secure them using M3 thin nuts (Item 15). Use two nuts per rod to prevent movement, or alternatively, use a single locking nut.
5. The direct coupling centrifugal water pump (Item 8) is held onto the backing board using the 3d printed motor lid (Item 3) using two M3 x 20 mm cap screws (Item 13) and two M3 thin nuts (Item 15).
6. Fix the flow meter (Item 10) onto the backing board using four (Item 14) M3 x 30 mm cap screws. The screws should lock into the nuts that are included in the flow meter assembly. Before mounting ensure that the supplied 1 mm nozzle jet is inserted into the inlet of the meter, this will ensure that the meter is configured to read between 0.05–0.5 L/min accurately. If your experiment requires faster flow rates, please read the technical specifications first as this is a very difficult procedure to reverse due to the push/tolerance fit.
7. Attach the two needle valves (Item 9) by first removing the T-handles with a hex key. Remove the compression nut to expose the ⅜” BSP thread. Screw the valve bodies into the backing board, ensuring the flow orientation matches the engraving. Replace the compression nut to secure the valve in place. To prevent the body from rotating during operation, secure it via the two M3 tapped holes on the backing board using suitable grub screws. Note: Acrylic thickness can vary between suppliers; counterbore the plate if the threaded section on the valve body is insufficient for a secure fit.
8. Plumb the components together once all units are attached. Use silicone flexible tubing (6.3 mm ID, 9.5 mm OD) for general connections. For the sections between Item 9–10, Item 9–Water Supply, and Item 7–8, use high-strength transparent PVC flexible tubing to prevent expansion or rupture under pressure. Secure all water connections that are not compression fits with appropriately sized hose or jubilee clips. Connect the flexible tubing from the needle valves to the Stirred Tank and Head SA via the straight and elbow push-fit adapters (Items 20 and 15 in drawing: MOSTR_Stirred_Tank_and_Head_SA_1.pdf).

#### 5.3 Control unit SA & system wiring

##### 5.3.1 3D printing

The control unit is made from a number of 3d printed parts as detailed in drawing MOSTR_Controller_Box_SA_1_Sht_1-5.pdf, all parts referred to were made with standard PLA filament and 0.4 mm nozzle arrangement. Following these printing guidelines:

● Print the Casing Back (Item 1, MOSTR_Casing_2_-_Back.stl) on its back face with supports used for the conductivity probe opening section.
● Print the Casing Front (Item 2, MOSTR_Casing_2_-_Front.stl) on its front face, with support from the bed.
● Print the Casing Base (Item 3, MOSTR_Casing_2_-_Base.stl) from the bottom face, no support required.
● Print the Controller box frame support (Item 4, MOSTR_Controller_Box_Frame_Support_1.stl) on its side with minimal support on the central plane.
● Print the Lego technical back boards (Item 6, MOSTR_Lego_Technical_Back_Board_1.stl & Item 5, MOSTR_Custom_Lego_Technical_Back_Board_1_-_M5_holder.stl) with the back flat to the bed and no supports.

Install M3 Brass Threaded Inserts into Items 1 and 3 as shown in the SA drawing. To do this, use a soldering iron to heat the inserts while applying light pressure until they fuse into the mounting holes of the 3D-printed material.

##### 5.3.2 Wiring

**Fig. 10:**
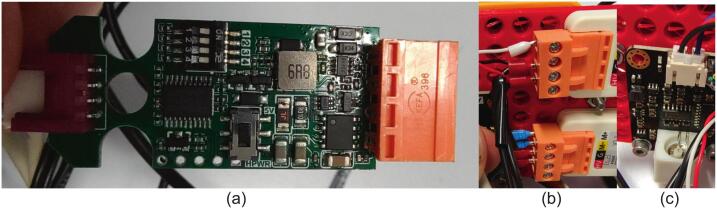
(a) HBridge internal circuitry showing the voltage selection microswitch, (b) HBridge cable connections, (c) Conductivity sensor control board wiring.
3. The motor/encoder unit comes with a premade cable with plug that has 6 short lengths of wires. To reach the control unit they are similarly attached to a screened multi-core cable (0.22 m^2^ conductor area) using heat shrinkable solder sleeves. The wires connect to the M5Stack HBridge that is connected to channel 1 of the M5Stack Expansion Unit (Itself linking to channel 0 of the M5Stack I2C hub) and to the M5Stack Encoder that is linked to channel 2 of the M5Stack Expansion Unit. The motor/encoder white (M1) and red (M2) wires are connected to the M- and M + respective connections of the HBridge unit. On the Encoder; Blue (GND) to Ground, Black (VCC) to 5 V, Green (C1) to B and Yellow (C2) to A. The connections from the 5.1 V DC 3A transformer are connected to the HBridge HV and G terminals (Note: set the internal microswitch to 5 V, see Fig. 10a). The motor side wires and connections are again covered in heat shrink
4. Channel 3 of the M5Stack Expansion Unit has a connector modified with the red, white and yellow wires and capped and the black earth connection soldered to a sheathed grounding wire that is connected to a known earth bonding plug.
5. The Conductivity sensor control board comes with a wired plug connector, this requires splicing with a conventional M5Stack 4 strand wire using solder sleeves. The wiring arrangement is blue to white, red to red, black to black, with the yellow cable not in use (See Fig. 10c). The cable should connect to the M5Stack 6-channel expansion Unit with I2C in channel 2.

Note: All wires that are connected to screw terminals are capped with crimpable ferrules with sizes appropriate to the wire gauge.

The main wiring in the system, as seen in Fig. 6, uses pre-made M5Stack four-wire connectors. The exceptions are as follows:

1. Strip back the sheath of the flow meter cable then attach to the M5Stack encoder (connected to channel 4 of the M5Stack I2C hub) by screw terminals. Red (+4.5 to 25 V) to 5 V, Blue (Signal) to Z and earth (Braid) to G.
2. The pump unit comes with 5 short wires, not all of which are required for the build. To reach the control unit they are attached to a screened multi-core cable (0.22 m^2^ conductor area) using heat shrinkable solder sleeves for rapid assembly and convenience. The wires connect to the M5Stack HBridge that is connected to channel 1 of the M5Stack I2C hub. The pump red (VCC) and green (CW/CCW Motor Rotation) wires are connected to HV, the black (GND) to the ground and the grey (VM Motor Power) to the M+, the motor yellow wire (RPM Pulse) is not connected. The HBridge side connections are also combined with the wires from the 12 V DC 1A transformer, with the positive linked to the HV and the negative to the ground. Important: Toggle the microswitch inside the M5Stack H-Bridge to 'high voltage' mode to ensure compatibility with this power supply (see Fig. 10). The pump side wires and connections are covered in heat shrink to avoid any bare wires and help waterproof.

##### 5.3.3 Assembly

The wire routing is shown in Fig. 6, the different hardware modules are mounted/orientated as shown in drawing SA MOSTR_Controller_Box_SA_1_Sht_1-5.pdf using Lego Technic Connector Pegs, the only exception to this M3 hex screws, washers and nuts used to attach the M5Tough and the Conductivity Meter Board.

Assemble the internal framework as follows:

1. Place the lower Lego Technical Back Board (Item 6) on to the Casing base (Item 3) as shown in sheet 4.
2. Attached the two Frame Supports (Item 4) to the back board (Item 6), they should clip into place with the retaining lip.
3. The second Technical Back Board can then be slid into the angled slot of the frame support as shown in sheet 2.
4. The Custom Technical Back Board (Item 5) with the cutout can then be slid over the M5Tough unit and snapped into place.
5. Align all cables with the two notches on the rear of the Casing Base (Item 3), then insert the Casing Back (Item 1) into the base. Clip the back into place via the two retaining ridges at the front and rear of the base and secure it using M3 screws driven into the threaded inserts at the rear of the base section.
6. Complete the assembly by sliding the Casing Front (Item 2) inside the Casing Back/Base. Secure it in place using the four M4 screws driven into the remaining four threaded inserts.

The conductivity probe can then be connected to the CU through the cutout in the casing with the bayonet connector, the type K thermocouple placed into the thermowell of the reactor vessel lid and the motor/encoder plugged in. The final connection is the USB cable, which can just be to a power supply or a PC for programming purposes.

#### 5.4 Compiling and uploading the program

Below is a simple, step-by-step guide to clone, build, and upload this project to an M5Tough using Visual Studio Code and PlatformIO. These are cross-platform tools that should work on Windows, Mac, and Linux.

1. Install the required tools (one-time)
● Visual Studio Code: https://code.visualstudio.com/
● PlatformIO IDE extension for VS Code:
  ○ In VS Code → Extensions (Ctrl + Shift + X) → search “PlatformIO IDE” → Install
  ○ Extension page: https://marketplace.visualstudio.com/items?itemName = platformio.platformio-ide
● Git:
  ○ If not already installed on your operating system, install from the official site:
  ○ https://git-scm.com/install/
● USB driver (M5 devices commonly use Silicon Labs CP210x):
  ○ CP210x VCP Drivers: https://www.silabs.com/developers/usb-to-uart-bridge-vcp-drivers
● Hardware
  ○ The M5Tough comes with a power/data cable that connects to a USB port.
2. Get the code from GitHub:
● git clone YOUR_REPO_URL
  ○ Replace YOUR_REPO_URL with your repository’s HTTPS or SSH URL (which always ends in “.git”):
● cd Modular-Open-Source-Stirred-Tank-Reactor
● code.
3. Let PlatformIO set up the project
● When the folder opens in VS Code, PlatformIO will read platformio.ini and automatically download the ESP32 toolchain, Arduino framework, and any declared libraries.
● The first setup can take a few minutes. You’ll see progress in the VS Code “Output” panel from PlatformIO.
4. Connect and power the M5Tough
● Plug the M5Tough into your computer via USB‑C.
● If it looks off, short-press the power button to wake or long-press (∼2s) to turn on.
● Find the COM port
  ○ On Windows: Device Manager → “Ports (COM & LPT)” → note the new COM port (e.g., COM5).
  ○ On Unix: Determine the serial device (e.g. /dev/ttyUSB0) by whatever means your operating system provides
5. Build the firmware
● In VS Code: click the PlatformIO toolbar checkmark (Build), or run in the terminal:
● pio run −e m5stack-tough
6. Upload (flash) the firmware to the M5Tough
● In VS Code: click the PlatformIO toolbar right-arrow (Upload), or run in the terminal:
● pio run −e m5stack-tough −t upload −-upload-port COM5
  ○ Replace COM5 with your actual COM port on Windows or the relevant serial device on Unix. PlatformIO compiles and then uses esptool.py to flash over USB.
7. View logs (Serial Monitor)
● In VS Code: PlatformIO → Monitor, or run in the terminal:
● pio device monitor −b 115,200 −-port COM5
  ○ Replace COM5 with your actual COM port on Windows or the relevant serial device on Unix.
  ○ 115,200 matches monitor_speed already in platformio.ini.
8. Optional: upload files to SPIFFS (if your project uses a data/ folder)
● pio run −e m5stack-tough −t uploadfs −-upload-port COM5
  ○ Replace COM5 with your actual COM port on Windows or the relevant serial device on Unix.

#### 5.5 IoT infrastructure

1. Install Docker
  ○ Linux: install docker engine https://docs.docker.com/engine/install/
  ○ Windows/Mac: install docker desktop https://www.docker.com/products/docker-desktop/
  ○ (Other docker clients you already use should work equally well)
2. Get the code from GitHub
  ○ Follow instructions in section 5.4
3. Update config files
  ○ In docker-compose.yml, update the value of DOCKER_INFLUXDB_INIT_PASSWORD to a suitably secure password
  ○ In the same file, update the value of GF_SECURITY_ADMIN_PASSWORD to a different password
4. Start the containers
  ○ Open the infrastructure directory from the repo in the command line
  ○ Run the command *docker compose up*
  ○ If you want to have the containers keep running after the end of your terminal session, instead use the command *docker compose up −d*
5. Set up Home Assistant MQTT integration
  ○ MQTT integration can only be set up in the UI. Open *http://<your server url or ip address>:8123* (if you are running it locally on your mac/pc, your server url will be *localhost*)
  ○ Follow the one-time setup instructions to create a username and password
  ○ Navigate to settings > devices & services
  ○ Search for ‘MQTT’ and select the option to add the service
  ○ Set the broker to *mosquitto*. Leave port as 1883, and username and password blank
6. Generate InfluxDB API tokens
  ○ Open the InfluxDB interface at http://<your server url or ip address>:8086
  ○ Enter the username *admin. The p*assword is whatever you set in step 3 (admin123 by default)
  ○ From the left-hand menus, find API Tokens
  ○ Generate one ‘all access’ API token called ‘Home Assistant’ and note it down somewhere temporarily
  ○ Generate a second ‘custom’ API token called ‘Grafana’, and give it read-only access to all buckets
7. Add InfluxDB API token to Home Assistant
  ○ In the config file found at */infrastructure/homeassistant/secrets.yaml*, add the Home Assistant API Key generated above in place of *YOUR_INFLUXDB_TOKEN_HERE*
8. Add InfluxDB API token to Grafana
  ○ In the config file found at */infrastructure/grafana/datasources/automatic.yaml*, add the Grafana API Key generated above in place of YOUR_INFLUXDB_TOKEN_HERE
9. Restart Containers
  ○ containers are stopped with ctrl + c if not started with the −d flag, or by running docker compose down from the same directory the containers were started with if started with the −d flag.
  ○ Start the containers again with the same docker compose up command as before (step 4)
10. Send a test MQTT message
  ○ Navigate to http://<your server url or ip address>:8123/config/integrations/integration/mqtt
  ○ Click the cog icon to open the MQTT settings screen
  ○ Publish a packet with the following topic and payload
    ■ homeassistant/sensor/test/sensor/config
    ■ {“name”:“test flow_sensor”, “uniq_id”:“test_sensor”, “stat_t”:“homeassistant/sensor/test/state”, “dev_cla”:“water”, “val_tpl”:“{{ '{{ value_json.val | is_defined }}' }}”, “device”: {“name”:“Test Device”, “identifiers”:[“test_device”]}}
  ○ Publish a second packet with this different topic and payload
    ■ homeassistant/sensor/test/state
    ■ {“val”: 1.23}
11. Verify this worked
  ○ Check the test dashboard in Grafana for data
    ■ Open Grafana at *http://<your server url or ip address>:3000*

Navigate to Dashboards > Default > Test Dashboard

■ You should see an entry in the table with value 1.23
■ If this is not the case, then try the next test. If that works, Grafana is not correctly configured to connect to InfluxDB. Check the API Token is correct
  ○ Check InfluxDB received the data
■ Open InfluxDB at *http://<your server url or ip address>:8086*
■ Navigate to Data Explorer
■ Construct a new query with two filters: domain = sensor and_field = value
■ With View Raw Data selected, one row we be displayed
■ If this is not the case, try the next test. If that works, Home Assistant is not correctly configured to sync to InfluxDB. Check the API Token is correct.
  ○ Check the device registered in Home Assistant
■ Open Home Assistant devices dashboard at http://<your server url or ip address>:8123/config/devices/dashboard
■ A device called Test Device will then be visible. Entering that device should show a sensor with a value of 1.23
■ If this is not the case, then there is something wrong with Home Assistant’s MQTT configuration

#### 5.6 Grafana

Each MOSTR unit has its own custom Grafana Dashboard, complete with a Canvas panel and four separate graphs for Water Temperature, Water Conductivity, Stirrer and Input Flow rate. The Grafana instance provisioned in Software/infrastructure/grafana of the https://doi.org/10.17632/z24ggxmg5f.3 repository (Latest at: https://github.com/University-of-Sheffield-MEE/Modular-Open-Source-Stirred-Tank-Reactor) comes with a dashboard featuring these panels. A text entry box at the top of the dashboard allows the inputting of the device id for which the dashboard should display data.

### 6 Operation instructions

#### 6.1 Pre-operation checks

Before powering or connecting the system:

● Verify all water connections are fully secured and free of leakage.
● Ensure the reactor vessel, tubing, and fittings are clean and flushed to prevent contamination.
● Position the vessel within the bund tray to contain accidental overflow.
● Inspect electrical cables to ensure they are kept clear of wet areas.
● Confirm the stirrer assembly (baffles and Rushton turbines) is present and correctly positioned (Fig. 3).
● Safety Notice: Water and powered components are in close proximity. Maintain dry cable routing and ensure bench areas remain free of spills. See “MOSTR_Risk_Assessment.pdf” in the https://doi.org/10.17632/z24ggxmg5f.3 repository under Design Files > Safety for further guidance.

#### 6.2 Probe calibration

Immediately after powering up the control unit, the conductivity probe calibration procedure will be triggered. The probe code is built upon the DFRobot DFR0300-H K = 10 Arduino example, adding a visual touchscreen interface rather than relying on a serial output for the process.

**Fig. 11:**
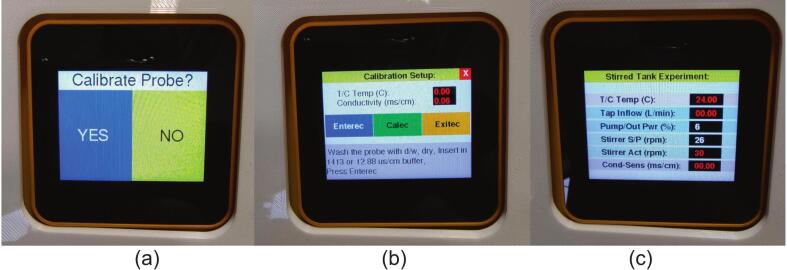
The various sequential interface screens during the calibration process and the operation screen. (a) calibration decision screen, (b) calibration setup screen, (c) “Stirred Tank Experiment” operation screen.
● Clean the conductivity probe with fresh distilled water, dry it and then place it in the readied calibration standard (12.88 µs/cm buffer, included in the probe kit). The calibration process is temperature controlled, but the thermocouple is sensitive to extremes of conductivity in the standard solution. To get an ambient water temperature reading, place the thermocouple in a sample of fresh tap water that has been left to equilibrate to match the standard’s temperature.
● The Calibration Setup screen will show next (Fig. 11b), with live readings of both the thermocouple and the conductivity probe at the top in red. There are also four touch sensitive buttons, “Enterrec” in blue, “Calec” in green, “Exitec” in orange and a red “X” in the top right corner, which is used to exit the process.
● The instructions of each stage of the calibration are given in the grey text box at the bottom of the Calibration Setup screen, following them will result in a proper calibration of the probe. If the calibration fails at any stage a “Failed, Try Again” message will display in the text box area. Note: If the control box loses power at any point, the calibration process will need to be repeated as the calibration value is reset upon the bootup process.
● The conductivity probe can then be inserted into the probe housing on the valve control panel, ensuring that the O-ring is tight to avoid leaks and the probe loop aperture is in parallel to the water flow.

● The calibration decision screen loads first (Fig. 11a) with the option “Calibrate Probe?”. The “YES”/”NO” sections on the display are touch sensitive, clicking on “NO” will take the controller in the operating state. Click on “YES” to start the calibration process.

#### 6.3 User Interface, stirrer motor and pump control

After the calibration setup screen has been completed or bypassed, the main “Stirred Tank Experiment” screen will load (see Fig. 11c), giving live control of the experiment. Each section is colour coded together for the relevant sensors and set points:

● T/C Temp (°C): Is a live feed of the water temperature of the vessel (via the thermowell).
● Tap Inflow (L/min): Shows the water flow rate into the stirred tank system in (L/min).
● Pump/Out Pwr (%): Gives a live feed of the % of power that is being sent to the water pump on the tank output. At 0 power the tank will drain only as fast as the head of water allows passively. Any % increase will increase the output flow, allowing a greater input flow rate to be achieved in the experiment whilst manually maintaining the tank volume with the needle valves. The value for this parameter is set using the labelled potentiometer on the front of the control box.
● Stirrer S/P (rpm): The field displays the stirrer speed setpoint for the stirred tank system. The value is set by using the labelled potentiometer on the front of the control box. The software PI control will attempt to adjust the stirrer motor to the value specified automatically.
● Stirrer Act (rpm): This field displays the actual real time stirrer speed value. Eventually this should get close to the S/P in the field above.
● Cond-Sens (ms/cm): This field displays a real time value of the conductivity value from the output stream of the stirred vessel.

#### 6.4 Calibrating the flow meter (recommended)

The water flow meter used in the project (RS 508–2704) theoretically measures correctly as a calibrated instrument. However, due to the compact nature of the control board there is a high likelihood that the readings will be subject to a systematic offset (measurement bias). To compensate for this, and the fact that the bias will be individual to each unit, a calibration coefficient has been included in the persistent config.json that is part of the file system image (see section 5.4). The “flowK”: 17000.0 value is the original ‘K’ factor for the 1 mm jet as per the unit’s data sheet, the “flowCorrectK” variable is a linear correction coefficient that is provided to compensate for any systematic flow rate discrepancy. The formula that governs the calibration is Q=f·60/k, where Q = flow rate in (L/min), f = the Hall-effect pulse frequency and the adjusted flow coefficient is k=60/K (where K≈17000).

To calculate this flow adjustment coefficient, perform the following procedure:

1. Take 6 readings of constant flow with the system, ranging from 0.1 to 1 L/min and record the average digital readout for each and the actual flow rate with a measuring cylinder and timer. Use a 5-litre total volume or greater to minimise the relative measuring uncertainty associated with manual timing and meniscus reading.
2. Plot the stock meter readings on the Y-axis and the actual measured readings on the X-axis. Perform a linear regression with a zero-intercept using a software package such as Excel. Use the resulting gradient value as the 'flowCorrectK' parameter in the software configuration.

The “flowCorrectK” value can then be added to the config.json file and uploaded to the M5tough unit over USB, following the restart the unit will be calibrated. If at any time you wish to go back to default values again, simply adjust the config.json “flowCorrectK” value back to 1 and repeat the calibration procedure. Perform this calibration if any adjustment to the pipework has been carried out.

#### 6.5 Flow balancing

The water feed is taken from a supply to the compression fitting on the inflow needle valve. If running an experiment with a single tank this would typically come from a pressure regulated lab tap with a jubilee clip, for reactors in series (see section 7) this would come directly from the outflow needle valve of the tank “upstream”. The inflow valve is used to adjust the flow rate into the tank during operation, the volume of water is kept constant in the vessel by manually balancing the input flow to the output flow. As the inflow rate is measured using the flow meter on the valve board, if the system is balanced, inflow and outflow will be the same. The system is designed to require continuous manual input, to simulate an industrial calibration scenario typical for continuous reaction processes in industrial chemical engineering.

The initial setup procedure is as follows:

1. Fully close both needle valves and fully open the water feed to the system (adjust the supply pressure regulator if necessary).
2. Gradually open the inlet valve and allow the water to enter to the desired fill level, then close it again.
3. Open the output flow control valve fully with the pump set to zero.
4. Gradually open the input valve again until the tank vessel is balanced at the desired fill level, when this has occurred, the control volume for the experiment is at steady state. If this is not possible and the tank is still filling, increase the pump power until balance is achieved.
5. The probe adapter and other pipework then need to be checked for any air bubbles. They can typically be released by inverting the equipment or removing the probe and reinserting.

Flow experiments can then be run at different flow rate by adjusting the valves and pump in the following way:

1. Set the input flow rate desired using the input need valve (using the control screen as a guide).
2. Adjusting both the output valve and pump as required until the control volume is back at the desired fill level.

#### 6.6 Tracer solution setup/injection

The tracer for the experiment is made by dissolving 65 g of NaCl in 200 ml of water to make a 5.56 M solution. To give a visual indication of the flow dilution process, food colouring or commercial tracers such as Fluorescein, Rhodamine etc can also be used.

When the flow rates are balanced as required, 20 ml of this tracer solution is injected via a syringe with a blunted tip into the control volume for each time run. The hole in the vessel lid is used as the injection point. The overall tracer dilution gives an initial concentration of 0.109 M NaCl in the vessel.

#### 6.7 Data streaming & data download

As mentioned in 2.6, 5.4 onward, data from all the main parts of the experiment is set up to stream automatically using IoT protocols. The data is organised on an instance of Home Assistant running on a VM and stored on the associated InfluxDB database. To access the streamed data and see a live stream of values a Grafana dashboard is required with the appropriate Query scripting.

**Fig. 12:**
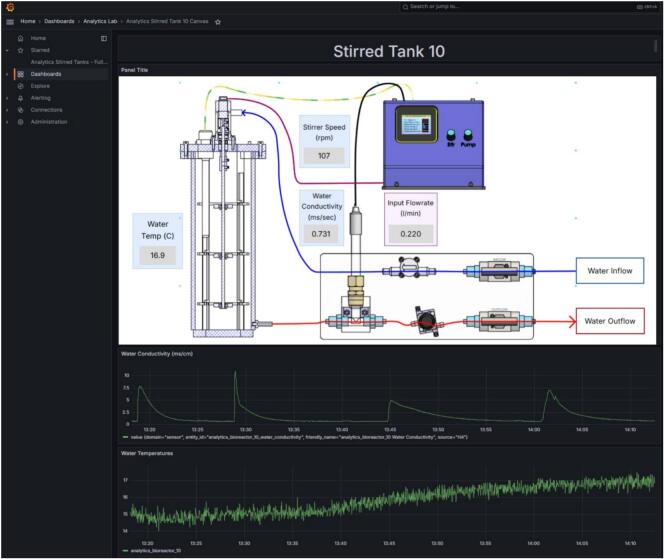
Grafana Dashboard Interface Example.

Each experiment rig has its own custom dashboard that is used to monitor live data (Fig. 12), this is broken down into the following sections:

● The Canvas Panel − This shows the last reading of each sensor with a diagrammatic image of the equipment.
● The Sensor Graphs − These show the data that has been logged over the time period selected.
● Data range − In the top right corner, there is a drop-down selection box for “local browser time”. This allows the data that has been recorded within a time range. i.e. over the last two hours, to be displayed.
● Data refresh frequency − In the top right corner, there is another drop down selection box for *“refresh time”*. This allows adjustment of the global refresh speed of the various sections on the dashboard.

Historic data is collected from each of the sensor graphs and downloaded for further analysis using the following process:

● On any of the graph panels, hover the cursor over the top right corner and three dots will appear, click on them and a menu will appear. Go to the option for “Inspect” −> “Data”.
● The data inspection menu will then appear showing all of the sensor data that has been logged during the time region previously specified along with the associated timestamp. The data is available to download in a spreadsheet compatible form by clicking on the “Download CSV” button in the upper right.

#### 6.8 End operations

● After all experiments have been completed, switch off all power to the experimental apparatus. Disconnect the two power supplies and the USB connection.
● Close the water supply and open all needle valves fully to relieve pressure.
● Remove the lid from the vessel and pour the remaining water down the sink.
● Leave the vessel to dry with the lid off or (preferred) use tissue to remove any residual moisture.
● Purge tubes and connectors of water to avoid stagnancy issues.
● Following the experiment, remove the conductivity probe, rinse it with DI water and dry. Store the probe in the supplied cage to prevent damage.
● Clean out the pipes and vessel with warm soapy water to ensure no future contamination. Avoid using isopropanol, as it will damage the acrylic parts.

#### 6.9 Troubleshooting

A list of common issues that occur during operation have been summarised in Table 4.

**Table 4:** Common issues and potential solutions for operation of the MOSTR system.

Problem and possible causes	Potential solution(s)
Conductivity reading is not accurate. ● The calibration procedure has not been run correctly. ● The earth connection is not sufficient. ● There is air trapped around the probe.	● Run the calibration procedure again. ● Ensure the thermocouple is reading a temperature that corresponds with the standard employed. ● The standards can get diluted over time due to residual moisture from the probe DI cleaning and get a new one periodically. ● When the water is flowing through the system at the desired rate, slide the probe from the enclosure and allow the coupling to overflow then re-insert the probe. ● Ensure that the probe sensing surfaces are parallel to the flow, not perpendicular. ● With water flowing, disconnect the earth connection and reconnect it while watching the conductivity reading. If there is no change, it is likely that there is a continuity issue in the earth wire or the earth is insufficient. Try earthing to the lab water supply.
The M5tough screen will not show. ● The power to the system is insufficient. ● The program has not been flashed onto the control board correctly. ● During assembly the internal M5tough connector pins are not seated correctly.	● Try connecting the M5tough USB connection to another power supply or a computer. Sometimes if the system is left connected and simply switched back on at the socket, the system will not boot until removing the USB and reconnecting. ● Connect the M5tough to a laptop with the VS Code platform IO software configured and view the serial output. If it is showing the program is running correctly it could be a loose connection with the LCD hardware. If there is no output, try re-flashing and uploading the code again. The serial output and VS Code compiler are excellent at giving error feedback, using any message shown to perform an online search and giving code snippets to LLMs like Google Gemini and ChatGPT so they can help with the diagnosis.
The pump is having little effect or the output flow rate. ● There is air inside the pump and/or it is cavitating.	● Ensure that the water is flowing throughout the system by gravity head alone and turn the pump power to 100 %. Angle up the control board 45 degrees but ensure that the pump is below the water line in the tank, this should allow the pump to prime using the air's natural buoyancy to remove it. The pump's sound should change noticeably during this process if air was the issue.
Parts of the control system seem not to be working. ● Defective components ● Bad wiring	● Many of the M5Stack modules have inbuilt power LEDs, these are inside the casings but are visible in a dark environment. Additionally, the motor encoder also has a LED power light. These are indicators of a proper connection, alongside serial line software feedback and debugging. ● Perform continuity checks on any wires that are leading from systems that are not functional, suggest starting the main I2C connection and following the “tree” down to the multiplexers systematically.
The flow rate measured is inaccurate. ● The incorrect jet insert has been used with the meter. ● The k factor calibration adjustment has been performed incorrectly. ● The meter is blocked.	● The flow meter (RS 508–2704) should have a 1 mm jet piece installed to work correctly with this system. ● Follow the steps again in section 6.4. ● Remove the tube around the flow meter and look to see if any debris is clogging the unit. If so, gently remove it without damaging the jet insert,
The stirrer rpm is very slow. ● Defective bearing. ● Misalignment of shafts. ● Shaft seal requires lubrication.	● Remove the motor coupling and motor and try to rotate the shaft manually, if there is resistance it could be indicative of any of the suggested probable causes. ● Remove the bearing and shaft seals and see the effect on the stirrer speed. If a watertight experiment is not required, removal of the shaft seal will enable the system to achieve the highest possible speeds of the geared motor. If keeping the seal, add a suitable lubricant to the inner shaft interface. ● If there is a miss alignment the bearing assembly loosen and reseat the shaft before retightening and testing. If the problem persists, drilling larger some of the non-tapped holes can help give more play for re-alignment.
The MOSTR system is not showing in Home Assistant. ● No discovery MQTT message is being transmitted. ● The device is not connected to the wi-fi. ● The config.json file has not been updated with the correct details.	● Check the serial output of the system to see if the discovered message is being transmitted correctly. ● Ensure that the config.json file has the correct wi-fi credentials and if multiple systems are running individual names, not all the same. ● In home assistant, use the listen functionality to see if IoT data packets are being received.

### 7 Validation and characterization

Each MOSTR system was tested and calibrated before use in the teaching laboratories, the typical capabilities of the design are described below. Note: each build will be slightly different in terms of performance, depending on assembly tolerances and quality of fabrication. However, broadly the data provided should give an indicative operational window to allow assessment of applicability to a range of reactor-based experiments, see the “Test Data” folder in repository https://doi.org/10.17632/z24ggxmg5f.3.

#### 7.1 Stirrer performance

**Fig. 13:**
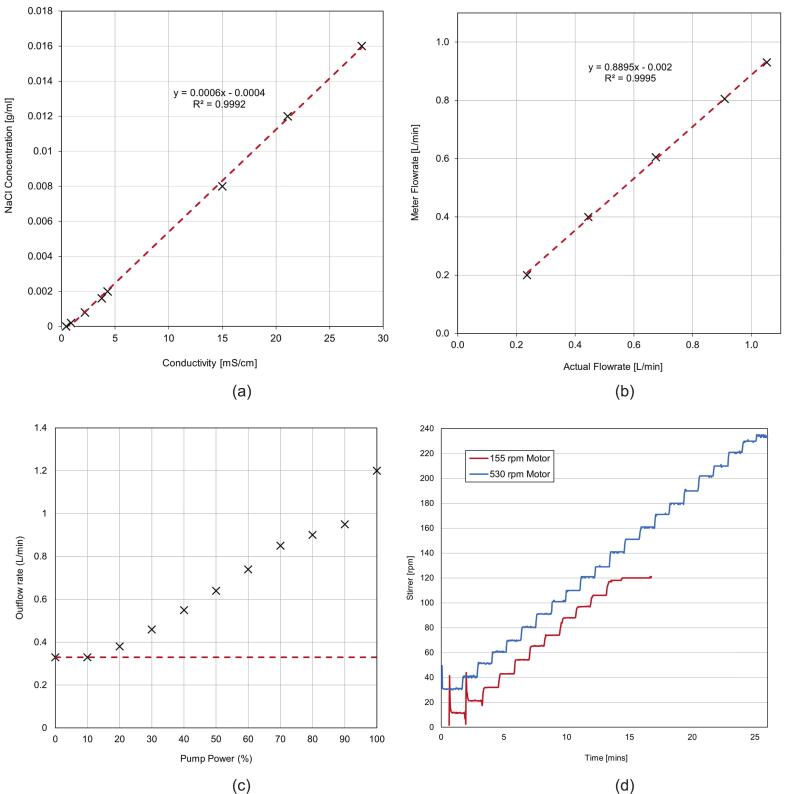
Calibration and characterisation data for the MOSTR system: (a) Conductivity probe calibration; conductivity value against varying tracer concentrations, (b) Example flow meter calibration; data sheet reading versus actual, (c) Pump power % against reactor flow, red line shows gravity dependant flow rate, (d) Stirrer rpm with two gearmotors against time, showing consistency of PI control. (For interpretation of the references to colour in this figure legend, the reader is referred to the web version of this article.)

During operation the MOSTR tank mixing is controlled by setting an rpm set point value on the control interface with the appropriate potentiometer (as described in section 6.3). To test the control consistency a test was performed setting the stirrer rpm to a set point from 0 − max in increments of 10 and allowing a minute for the speed to stabilise (see Fig. 13d). The data shows the feedback performance is consistent across the range and the readings are consistent almost instantaneously after the control adjustment is made (the first 10 s of each “step”). Below 30 rpm, there is more variance on the rpm due to the limits of PWM control and motor friction; however, the average reading is more than accurate enough for experimentation. The upper limit of rotational speed for the system with the 155 rpm and 530 rpm gearmotors is approximately 155 rpm and 235 rpm respectively. Please note: the second 530 rpm motor data was collected whilst using the larger Ø 50 mm Smith impellers (see section 7.9.1), rather than Ø 26 mm Ruston so the fluid resistance is much higher. The raw data was collected using the IoT/Grafana interface and is available in MOSTR_Stirrer_Speed_Consistency_r2.xlsx.

#### 7.2 Flowmeter calibration

As described in section 6.4, due to upstream/downstream flow effects around the flow meter the calibration data produced based on factory defaults can lead to systematic errors (measuring bias). An example of this is included in MOSTR_Flowrate_Calibration_Example.xlsx, with the calculation to produce the correction coefficient by linear regression. The graph in Fig. 13b demonstrates a typical discrepancy.

#### 7.3 Conductivity probe salinity calibration

Although the MOSTR system has inbuilt calibration for the conductivity probe (section 6.2), the units themselves (ms/cm) do not directly correlate with the NaCl tracer solution (g/ml, section 6.6) used for the experiments. A representative set of calibration data showing this relationship is included in the repository: MOSTR_Conductivity_Probe_Calibration.xlsx. The associated linear regression relation: NaCl Conc (g/ml) = 0.0006 x Conductivity Reading (ms/cm) + -0.0004, is shown in Fig. 13a.

#### 7.4 Pump flow relationship

The MOSTR system is capable of operating without the outlet pump, although the flow rate within the vessel is then limited to gravitational flow. To quantify the impact of the pump on balanced flow throughput, a series of tests were conducted by increasing the pump power in 10 % increments while maintaining a constant hydraulic head of 0.2 m (see Fig. 13c). The unpowered flow rate is approximately 0.33 L/min, with the effects of the pump becoming measurable only above 20 % power. At 100 % power, the system achieves a maximum flow rate of approximately 1.2 L/min. Based on these data, it was determined that the pump provides sufficient head to operate in a recirculating configuration. The raw data collected via the IoT and Grafana interface are available in the supplementary file MOSTR_Pump_Flow_Relations.xlsx (under the Test Data/Calibration section of the software repository, https://doi.org/10.17632/z24ggxmg5f.3).

#### 7.5 Mixing performance analysis / characterisation approach

The MOSTR system was primarily developed to facilitate the teaching of experimental RTD characterisation in chemical reactor systems. For each experimental condition, the conductivity response to the tracer pulse was logged via the IoT framework to a Grafana dashboard. To ensure the integrity of this logged data for analytical purposes, it is necessary to distinguish between corrected systematic offsets and inherent random noise. While the calibration procedures outlined in previous sections effectively mitigate systematic errors (bias) across the sensor suite, experimental measurements remain subject to relative (random) errors that contribute to overall measurement uncertainty. Within the MOSTR platform, these sources of variance primarily include analogue-to-digital converter (ADC) quantisation limits, hydrodynamic fluctuations introduced by the stirrer and needle valves, and electronic signal noise from the conductivity probes—the latter being inherently linked to the quality of the system's earthing. To minimise the impact of these random variations on the final RTD calculations, the system utilises high-frequency data sampling and digital signal averaging directly on the microcontroller. Depending on the task and sensor combination, raw samples are captured every 25 to 1000 ms (as detailed in the main.cpp file of the software repository, https://doi.org/10.17632/z24ggxmg5f.3). Although the system's alternative Serial/WebRTC architecture is fully capable of much higher-frequency, low-latency data logging for advanced dynamic studies, the IoT-based 5-second averaged intervals are optimal for these specific educational CSTR experiments. Because the reactors operate under steady-state continuous flow conditions, transient micro-fluctuations are analytically negligible. This averaging approach thereby ensures that the aggregate uncertainty remains highly acceptable for pedagogical applications.

With these random uncertainties mitigated during data acquisition, the design efficacy of the MOSTR system was validated to ensure it provides a robust platform for student learning. A combination of the “Tanks-in-Series” (TIS) and “two-parameter” flow models was employed to identify and quantify phenomena such as flow stagnation and bypassing [8], [9]. These empirical results were compared with theoretical estimates of in-tank turbulence to confirm that the hardware design minimises non-ideal flow effects. By using these metrics to ensure the system’s performance is “good enough” for high-fidelity data collection, we guarantee that students can accurately observe and study distinct residence time differences without being obscured by system-induced artefacts. This rigorous characterisation ensures the MOSTR platform serves as a reliable tool for mastering experimental reactor analysis in a laboratory setting. Comprehensive details regarding these characterisation tests are available in the online repository under “Test Data/Experiments”.

##### 7.5.1 RTD, TIS and two parameter calculation

The following procedure was conducted to assess system performance using the tracer pulse technique:

1. For each logged time step (t*,* cumulative for the duration), the conductivity (mS/cm) was converted via the regression specified in Section 7.3 to determine the NaCl equivalent tracer concentration as a function of time ($C\left(t\right)$, g/ml).
2. As the inlet and outlet flow rates are balanced (i.e. the hydraulic head of the tank was kept constant), the amount of the tracer leaving the reactor was then calculated by Eq (1), where ΔN = Amount of tracer (g), v = volumetric flow rate (ml/sec) and Δt = time between measurements (sec).
3. The total amount of tracer used during the experiment $N_{0}$ is then calculated by integration of the outlet concentration curve, Eq (2).
4. The Mean Residence Time, $\overset{¯}{t}$, representing the average time the tracer spends in the reactor vessel was calculated from the Concentration versus Time data or the “C curve”, Eq (3).
5. The reactor Space Time, τ(or $\tau_{\mathrm{cuml}}$), the time required to process a reactor volume (one or multiple) of feed was calculated in Eq (4), where $v_{0}$ = inlet volumetric flow rate (ml/sec) and V (or $V_{i}$) = the reactor working volume (ml).
6. Performing a linear regression on a log plot of $C\left(t\right)$ against t (the tail of the tracer decay curve), the slope (S) is used to calculate the reactor Active Volume, α, using the following relation in Eq (5).
7. The Bypass flow fraction, β, is calculated in Eq (6), where M = Amount of NaCl injected in tracer (g).
8. The RTD, $E\left(t\right)$, per unit time is then calculated by Eq (7).
9. The $E\left(t\right)$ data was then used to calculate the statistical Variance, $\sigma^{2},$ distribution, see Eq (8).
10. To help with further results comparison a TIS one parameter model is calculated by making a dimensionless version of the Variance, $\sigma_{\theta }^{2}$, Eq (9), where n = Is the “ideal” tank TIS.
11. In the case of multiple vessels in series, β, α cannot be used for any of the reactors following the first because the response is not an exponential decay (actually a bell curve), so the regression would not work and Eq (9)’s n value becomes cumulative. To give another metric, Eq (10) is used to calculate a Total Volume Utilisation, Φ, which dimensionlessly compares the total experimentally measured residence time to the sum of the Space Time of all vessels in the chain. Where the value of 1.0 indicates perfect utilisation and a value < 1.0 indicates bypass or dead zones. $\overset{¯}{t_{i}}$ = mean residence time determined from the tracer curve at the outlet of the vessel at that point in the series.

The results of these equations are cross referenced against the following data metrics for performance comparison:

● TIS n values:
  ○ < 1: Bypassing (Short-Circuiting) combined with Dead Zones
  ○ ∼ 1: Ideal CSTR performance. Fluid enters and is instantly mixed into the entire volume.
  ○ > 2: Segregated Flow (Poor mixing for a CSTR, but potentially good if you wanted a Plug Flow Reactor (PFR). i.e. behaving like an infinite number of tiny mixing zones, which mathematically creates smooth flow with zero back-mixing.
● Active Volume (α) %: Ideal value is 100 %, showing the entire volume is in use.
● Bypass Fraction (β) %: Ideal value is 0 %, showing flow going through the vessel with no mixing.

(1) $$\Delta N=C\left(t\right)v\Delta t$$

(2) $$N_{0}=\int_{0}^{\infty }C\left(t\right)v\Delta t$$

(3) $$\overset{¯}{t}=\frac{\int_{0}^{\infty }t\Delta N}{N_{0}}$$

(4) $$\tau =\frac{V}{v_{0}}or\;\tau_{\mathrm{cuml}}=\frac{\Sigma_{i=1}^{N}V_{i}}{v_{0}}$$

(5) $$\alpha =\frac{1-\beta }{S\tau }$$

(6) $$\beta =1-\frac{N_{0}}{M}$$

(7) $$E\left(t\right)\Delta t=\frac{C\left(t\right)v\Delta t}{\int_{0}^{\infty }C\left(t\right)v\Delta t}=\frac{\Delta N}{N_{0}}$$

(8) $$\sigma^{2}=\int_{0}^{\infty }{\left(t-\overset{¯}{t}\right)}^{2}E\left(t\right)\Delta t$$

(9) $$\sigma_{\theta }^{2}=\frac{\sigma^{2}}{\overset{¯}{t^{2}}}=\frac{1}{n}$$

(10) Φ=tfinalΣi=1Nrτi

##### 7.5.2 Impeller Reynolds, stirrer power and energy dissipation calculation

The following procedure was conducted to assess the system's mechanical mixing performance based on input parameters:

1. Under the assumption of water at 20 °C (Density ρ = 998.2 kg/m^3^ and Dynamic Viscosity μ = 1.002 x 10^-3^ kg·m^−1^·s^−1^), the Reynolds Number (Re) for the flow regime surrounding the impeller was calculated via Eq. (11). This calculation incorporates the impeller diameter $D_{i}$ (m) and the rotational speed c (rev per second).
2. The power transmitted from the impeller to the liquid (P, in Watts) was estimated using the empirically derived dimensionless Power Number ($N_{p}$), as shown in Eq. (12). This estimation accounts for external variables such as motor gear ratios and mechanical friction that preclude direct measurement. Similar to aerodynamic drag coefficients, $N_{p}$ is dependent on geometry and turbulence levels. Within the viscous-dominated laminar range, $N_{p}$ is governed by the relation in Eq. (13), where $K_{p}$ represents the laminar constant. Conversely, within the fully turbulent Re range, $N_{p}$ (specifically, $N_{p\infty }$) remains constant.
3. To facilitate a model capable of dynamically calculating $N_{p}$ across a broad Re range for various impeller geometries, the Churchill-Usagi correlation was employed (Eq. (14). This approach utilises a series of empirical coefficients for $N_{p\infty }$ and $K_{p}$, where m represents the transition factor used to weight the transition between the laminar and turbulent regimes.
4. The Average Energy Dissipation Rate, ${∊}_{\mathrm{avg}}$, is used to calculate the amount of energy being transferred into the fluid per unit of mass (W/kg) and gives an indication of the turbulence intensity, see Eq (15). This metric is a great comparator, as it can factor different volumetric scales and show differences in mixing intensity.

(11) Re=Di2cρμ

(12) $$P=N_{p}\rho {D_{i}}^{5}N^{3}$$

(13) Np-lam=KpRe

(14) Np=KpRem+Np∞m1m

(15) $${∊}_{\mathrm{avg}}=\frac{P}{\rho V}$$

The results of these equations are cross referenced against the following data metrics for performance comparison:

● Re values:
  ○ < 10: Laminar Flow
  ○ > 10 < 10,000: Transitional Flow
  ○ > 10,000: Turbulent Flow − Preferred regime for a CSTR.
● ${∊}_{\mathrm{avg}}$ Typical Values (W/kg):
  ○ Static Mixers: 10–1,000
  ○ Agitated vessels: Most applicable to CSTR 0.1–100.
  ○ High Speed Rotor-Stator: 1,000–100,000

#### 7.6 Single system experiment 1

**Fig. 14:**
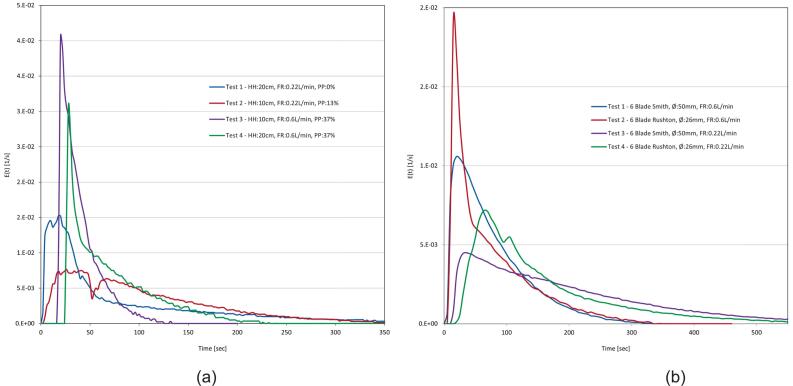
Residence Time Distribution (RTD), E(t), curves for a single reactor system across varying operational parameters of flow rate (FR in L/min) and tank hydraulic head (HH in cm) using the same high salinity tracer injection. The x-axis represents Time in seconds and the y-axis represents the RTD, E(t); (a) Single System Experiment 1: varying HH, FR with Ø26 mm Rushton impellers. (b) Single System Experiment 2 (section 7.9.1): comparing two impeller designs with different diameters (Ø26 mm Rushton and Ø50 mm Smith) at two flow rates with the same HH. Note: pump power (PP, %) provided for reference only.

To validate the MOSTR performance an initial test was performed varying Flow Rate (FR, in L/min) and Hydraulic Head (HH, in cm). Ø26 mm Rushton impellers were installed, the stirrer speed was set to 100 rpm and the same known tracer impulse was used each time (20 ml, see section 6.6). Test 1 − HH: 20, FR: 0.22, Test 2 − HH: 10, FR: 0.22, Test 3 − HH: 10, FR: 0.6, Test 4 − HH: 20, FR: 0.60. The subsequent RTDs (E-Curve) are shown in Fig. 14a below and summarised in Table 5; individual plots are available in the repository within MOSTR_Single_System_Residence_Data_1.xlsx.

**Table 5:** Experiment 1 − Overview of single MOSTR system mixing performance metrics. All tests were mixed at 100 rpm with a Rushton Impeller, producing a Transitional Re of 1140 and a subsequent $N_{p}$ of 5.

Test	HH (cm)	v(L/min)	Pump Power (%)	No Imp	P(W)	ε avg (W/kg)	$\underset{\_}{t}$(sec)	α (%)	β (%)	n
1	20	0.22	0	3	8.56E-04	8.90E-04	87.1	14.4	63.7	0.89
2	10	0.22	13	2	5.71E-04	1.19E-03	103.5	26.7	64.0	1.94
3	10	0.6	37	2	5.71E-04	1.19E-03	40.1	20.6	56.0	4.11
4	20	0.6	37	3	8.56E-04	8.90E-04	72.6	22.7	61.9	2.94

The results from experiment 1 were processed using the methodology described in section 7.5 and are summarised in Table 5. Under these conditions, the analysis characterises the current MOSTR system in a laminar-to-transitional regime (Re 1,140), where hydrodynamic performance is constrained by a specific power input (Max 0.001 W/kg) significantly below the threshold for ideal CSTR homogeneity. Experimental RTD across all four test conditions revealed a distinct non-ideal flow model dominated by high bypass fractions (β approx. 60 %) and substantial stagnant zones (1/α > 75 %), particularly in the high HH scenarios (Tests 1 & 4) where the input energy was most diluted. However, the system demonstrated a clear and positive sensitivity to operating conditions; notably, reducing the hydraulic head in Tests 2 and 3 effectively doubled the active volume fraction and improved the TIS characteristics (n approx. 4.1), confirming that the fluid dynamics are highly responsive to marginal increases in energy dissipation density. These results successfully validate the platform's operational stability and baseline functionality while identifying a precise engineering solution for future optimisation; the implementation of larger-diameter impellers to leverage ${D_{i}}^{5}$ power scaling (Eq (11). This initial hypothesis was subsequently tested in section 7.9.1.

#### 7.7 Systems in series residence time

To test the ability of the MOSTR system to study the effects of reactor staging on residence time distributions, three units were combined as a cascade of three CSTRs in series. This setup allows the experimental validation of the TIS model and the observation of the progression of mixing efficiency. Each system was operated at a HH of 20 cm and a v of 0.175 L/min. A tracer pulse was injected at the inlet of vessel 1 and the cumulative response was measured at the outlet of each stage (vessels 1, 2, and 3).

**Fig. 15:**
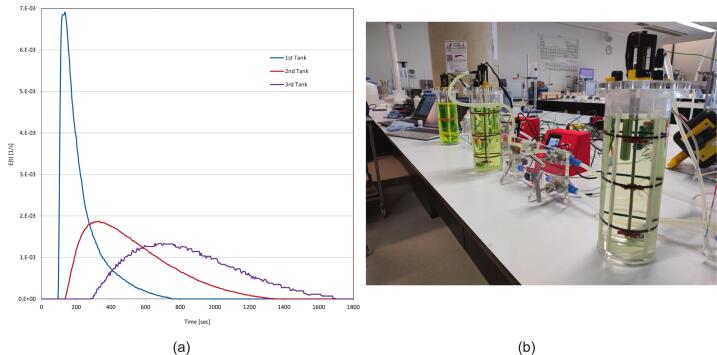
(a) Residence Time Distribution (RTD), E(t), curves for three MOSTR systems connected in series and each equally balanced with a flow rate (FR in L/min) and tank hydraulic head (HH in cm) of 0.175 L/min 20 cm respectively. The systems were all equipped with 3 Ø26 mm Rushton impellers set at a rotational speed of 100 rpm. The high salinity was injected into the first tank in series and allowed to propagate through the system. The x-axis represents Time in seconds and the y-axis represents the RTD, E(t). (b) Showing the sample experiment in progress, the intensity of the fluorescein tracer showing the periodic dilution process.

The resulting RTD curves (Fig. 15a) and visual colour change due to the fluorescein (Fig. 15b) provide a clear comparison between the single-tank and multi-tank responses. The progression from Vessel 1 to Vessel 3 illustrates the dampening of the tracer peak, visually demonstrating the principle of variance reduction in reactors with a series configuration, see MOSTR_Triple_System_Residence_Data.xlsx. Numerical analysis is presented in Table 5, demonstrating the cumulative evolution of the Total Volume Utilization (Φ) and TIS (n) number across the cascade (Re approx. 1140, $N_{p}$= 5).

The commissioning data establishes the MOSTR system as an effective platform for demonstrating experimental divergence between theoretical design and operational reality (Table 6). While the first vessel exhibits significant short-circuiting (Φ approx. 67.5 %), in keeping with the data in 7.6, the series configuration serves as a practical demonstration of how modular scaling can recover system performance. As the fluid progresses to the third stage, the cumulative Total Volume Utilization (Φ) rises to 83.5 %, and the TIS value reaches approx. 8.2, effectively illustrating how series architecture forces greater volumetric engagement even in mixing-limited environments.

**Table 6:** Overview of three MOSTR systems in series mixing performance metrics. All tests were mixed at 100 rpm with 3 Rushton Impellers, producing a Transitional Re of 1140 and a subsequent $N_{p}$ of 5. The HH was maintained at 20 cm with a v of 0.175 L/min. As these parameters were fixed, P = 8.56E^-04^ W and ε avg = 8.90E^-04^ W/kg in each case.

Tank	$\underset{\_}{t}$(sec)	$\tau_{\mathrm{cuml}}$(sec)	α (%)	β (%)	n	Φ
1	225.1	333.6	15.8	84.2	3.73	0.67
2	515.1	667.1	−	−	4.71	0.77
3	835.5	1000.7	−	−	8.23	0.83

#### 7.8 Example class data

**Fig. 16:**
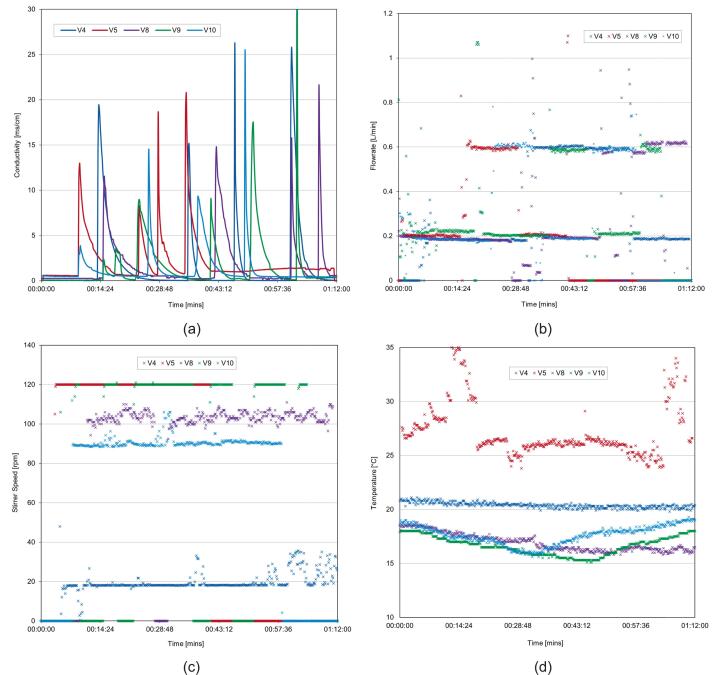
Example data collected during a 1 h laboratory teaching session using multiple MOSTR systems, where V# represents one system in standalone operation. In each graph the x-axis represents Time in minutes, the y-axis corresponds in each to: (a) Conductivity readings (ms/cm), (b) Flow rates (L/min), (c) Stirrer Speeds (rpm) and (d) Temperature readings (°C). Note: Each test was set up with a different HH variable, which was not recorded.

Ten MOSTR systems were successfully deployed for teaching the RTD concept in 2025, where students selected their own flow rates, stirrer speeds and experimental durations. The resulting data are presented in Fig. 16 (with the full dataset available in MOSTR_Teaching_Session_Data.xlsx). It is clear from the data collected that the tracer peaks (Fig. 16a) are highly distinct and the flow rates / stirrer speeds are consistent (Fig. 16b & 16c), which provide an ideal platform for experimental analysis and future experimental variation (i.e. change of impeller design, as highlighted in section 7.7). As the impeller rod is only retained by a single grub screw there is potential for the students to select the impeller design they will use or “hot swap” during future sessions.

##### 7.8.1 Single system experiment 2 − impeller effects on single system Residence time

**Fig. 17:**
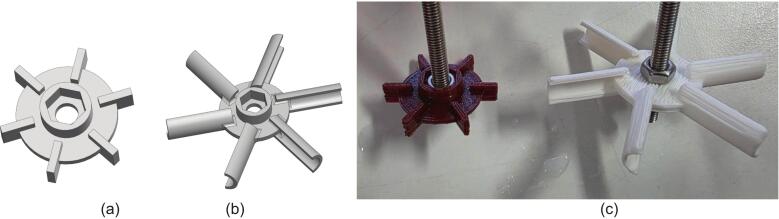
The two types of Impellers used in Single System Experiment 2; (a) CAD model of the 26 mm Ø Rushton impeller, (b) CAD model for the 50 mm Ø Smith impeller, (c) The actual respective 3d printed impellers as mounted on the stirrer shaft, for visual size comparison.

To explore the effects of varying impeller geometry on the MOSTR system, a second experimental series comprising four additional tests was conducted. These tests utilised flow rates of 0.22 and 0.6 L/min at a constant hydraulic head of 20 cm, comparing two distinct impeller designs as detailed in Table 7 (Data Source: MOSTR_Single_System_Residence_Data_2.xlsx). The first was the Ø26 mm Rushton turbine used in previous sections, while the second was a larger Ø50 mm 6-bladed Smith design (see Fig. 17). By increasing the impeller diameter at a fixed speed, the system leveraged $D_{i}$^^5^ power scaling to elevate the mean specific energy dissipation rate from negligible levels, ∼0.001 W/kg to ∼ 0.020 W/kg, a 22-fold increase. This effect was shown to successfully reduce the hydraulic bypass fraction (β) from 54 % to 40 % under high-flow conditions (0.6 L/min) and improved the internal circulation, as evidenced by an increase in the TIS value (n) from 1.4 to 1.8. However, despite these improvements, the persistence of significant stagnant zones (>50 % dead volume) indicates that while larger radial-flow impellers can mitigate short-circuiting, a single impeller stack cannot fully overcome the lack of top to bottom tank circulation when operating at Reynolds numbers not in the full turbulent regime.

**Table 7:** Single System Experiment 2 − Overview of single MOSTR system mixing performance metrics. All tests were mixed at 100 rpm, a HH of 20 cm and had 3 impellers.

Test	Imp Type	v(L/min)	Pump Power (%)	D_i_ (mm)	Re	P(W)	ε avg (W/kg)	$\underset{\_}{t}$(sec)	α (%)	β (%)	n
1	Smith	0.6	40	50	4151	1.91E-02	1.98E-02	77.4	43.7	40.1	1.78
2	Rushton	0.6	40	26	1140	8.56E-04	8.90E-04	76.9	37.1	53.6	1.41
3	Smith	0.22	−	50	4151	1.91E-02	1.98E-02	188.9	20.8	70.0	1.87
4	Rushton	0.22	−	26	1140	8.56E-04	8.90E-04	156.5	13.8	71.0	1.98

Beyond confirming its modularity, the tests again demonstrate that the system’s instrumentation possesses high sensitivity, capable of resolving subtle hydrodynamic shifts through bulk statistical analysis. In addition, the ability to provide instantaneous visual feedback, as evidenced by the distinct curve morphologies in Fig. 14b, across different impeller geometries and flow rates, firmly establishing the system's potential as a valuable experimental learning tool.

##### 7.8.2 Single system experiment 3 − stirrer motor gear-ratio effects on residence time

To evaluate the impact of elevated agitation rates on RTD, the reactor's primary drive mechanism was upgraded. The initial high-torque gearmotor (DFRobot FIT0483; 100:1 ratio, 1.5 kg·cm instant torque, 155 rpm no-load) was replaced with a higher-speed alternative (DFRobot FIT0481; 30:1 ratio, 0.45 kg·cm instant torque, 530 rpm no-load). The baseline configuration provided excess torque, but was mechanically speed limited by its gear ratio, plateauing at 120 rpm under standard fluid loads (Fig. 13d). By optimising the torque-to-speed ratio for the specific fluid drag of the system, the 30:1 gearbox achieved sustained operational speeds of 226–278 rpm (Table 8). The necessary software modifications to the PI controller and pulse frequency settings, accounting for the updated encoder resolution, are documented in the project repository (https://doi.org/10.17632/z24ggxmg5f.3, directory: Software/Motor modifications).

**Table 8:** Overview of single MOSTR system mixing performance metrics equipped with a 530 rpm gearmotor and three 50 mm Ø Smith impellers.

Test	HH (cm)	v(L/min)	Pump Power (%)	Motor speed (rpm)	Re	P(W)	ε avg (W/kg)	$\underset{\_}{t}$(sec)	α (%)	β (%)	n
1	20	0.2	0	229	9504	2.3E-01	2.4E-01	184.3	42.7	32.2	1.6
2	20	0.6	40	226	9369	2.2E-01	2.3E-01	64.8	33.4	41.0	2.2
3	12	0.2	−	278	11,539	2.7E-01	4.7E-01	110.9	36.3	41.0	1.7
4	12	0.6	40	259	10,737	2.2E-01	3.8E-01	49.1	52.4	33.3	1.8

This increase in rotational velocity, coupled with the ${D_{i}}^{5}$ power scaling characteristic of the 50 mm Ø Smith impellers, yielded a substantial increase in ${∊}_{\mathrm{avg}}$. Values reached between 0.23 and 0.47 W/kg, successfully transitioning the MOSTR system from a lower-transitional flow state into a fully turbulent hydrodynamic regime (Re 9,369–11,539) see repository: MOSTR_Single_System_Residence_Data_3.xlsx. Crucially, this places the platform's mechanical power input well within the standard operational window for industrial and laboratory continuous stirred-tank reactors (0.1–100 W/kg) [10]. Operating in this fully turbulent regime resolved the primary mixing limitations identified in earlier commissioning tests (Sections; 7.6, 7.9.1). At a low volumetric flow rate (0.2 L/min), the enhanced turbulence improved entrainment of the tracer, reducing the hydraulic β from 70.0 % to 32.2 %. Whilst concurrently augmenting fluid circulation previously disrupted by stagnant zones, demonstrated by an increase in the α from 20.8 % to 42.7 %. Notably, despite these significant improvements in active mixing volume, the n remained consistent, ranging between 1.6 and 2.2. This stability suggests that the residual non-ideal flow characteristics could be a function of the vessel's geometry, rather than an artifact of insufficient mechanical agitation. However, from an educational and operational perspective, these results validate the modular architecture of the MOSTR platform and highlight its versatility.

##### 7.8.3 Future development

Future iterations of the MOSTR platform will focus on enhancing its hydrodynamic versatility, operational autonomy and hardware integration. Specific areas of development include:

1. Hydrodynamic Optimisation for Laminar Regimes: To effectively utilize the high-torque, low-speed characteristics of the original DFRobot FIT0481 gearmotor, future work will include the design and characterization of a wall-scraping anchor impeller ($D_{i}$/$D_{t}$ approx. 0.9). This geometric configuration is specifically intended to physically sweep the vessel walls and displace stagnant fluid, providing an ideal mixing solution for highly viscous or strictly laminar flow applications.
2. Electrical Architecture Consolidation: The current power distribution system will be streamlined. The requirement for three separate mains connections will be replaced by a unified power distribution circuit, utilising a single transformer to improve laboratory safety, reduce desktop footprint and simplify assembly.
3. Calibration Improvement: Include a series of calibration screens similar to the conductivity probe, to allow input of the flow meter k calibration coefficient.
4. Sensor Interface Refinement: To address issues with trapped air disrupting conductivity measurements, the probe housing will be redesigned to incorporate an integrated degassing port. This will allow users to purge air bubbles locally without needing to unseat or completely remove the probe from the fluid stream.
5. Full Automation and Remote Operability: The system’s control capabilities will be expanded to support fully automated, remote experimental workflows. This will be achieved by integrating continuous water level sensors, solenoid-actuated flow valves, an automated tracer injection module and webcam monitoring.

### Ethics statements

A parallel study associated with this work was reviewed and approved by The University of Sheffield Ethics Committee with the approval number: 067533, dated 16/04/2025. This research (associated with the design/construction of the MOSTR system) does not directly include elements associated with the 067,533 study, just the resultant data logged during the student’s tests. In the parallel 067,533 study, all participants were informed that consent to participate in the study and publish their data would be dictated by their completion and submission of the study consent form, all works described here are outside of this scope and exempt from needing ethical approval.

### Declaration of generative AI and AI-assisted technologies in the writing process

During the preparation of this work the author(s) used Chat GPT 5 and Google Gemini 3 Pro in order to assist in the summaries of code implementation workflows, assist in mathematically modelling, making improvement in language clarity and in conjunction with Visual Studio Code as a software coding assistant. After using this tool/service, the author(s) reviewed and edited the content as needed and take(s) full responsibility for the content of the publication.

### CRediT authorship contribution statement

**Krys Bangert:** Writing – review & editing, Writing – original draft, Visualization, Validation, Supervision, Software, Resources, Project administration, Methodology, Investigation, Funding acquisition, Data curation, Conceptualization. **Edward Browncross:** Writing – review & editing, Writing – original draft, Visualization, Validation, Software, Resources, Data curation. **Chalak Omar:** Validation, Investigation, Formal analysis, Conceptualization.

### Funding

This research did not receive any specific grant from funding agencies in the public, commercial, or not-for-profit sectors. Publication fees were covered by the University of Sheffield’s Institutional Open Access Fund. For the purpose of open access, the author has applied a Creative Commons Attribution (CC BY 4.0) licence to any Author Accepted Manuscript version arising.

## Declaration of competing interest

The authors declare that they have no known competing financial interests or personal relationships that could have appeared to influence the work reported in this paper.
